# Abnormal DNA methylation analysis of leucine-rich glioma-inactivated 1 antibody encephalitis reveals novel methylation-driven genes related to prognostic and clinical features

**DOI:** 10.1186/s13148-023-01550-5

**Published:** 2023-08-29

**Authors:** Shan Qiao, Quanye Sun, Haiyun Li, Jie Yin, Aihua Wang, Shanchao Zhang

**Affiliations:** 1 Department of Neurology, The First Affiliated Hospital of Shandong First Medical University and Shandong Provincial Qianfoshan Hospital, Shandong First Medical University, Jinan, China; 2 Department of Medical Genetics, School of Basic Medical Sciences, Cheeloo College of Medicine, Shandong University, Jinan, China; 3 Research Center of Translational Medicine, Central Hospital Affiliated to Shandong First Medical University, Jinan, China; 4 Department of Neurology, Qilu Hospital, Cheeloo College of Medicine, Shandong University, Jinan, China; 5 Department of Cardiology, The First Affiliated Hospital of Shandong First Medical University and Shandong Provincial Qianfoshan Hospital, Jinan, China; 6 School of Medicine, Cheeloo College of Medicine, Shandong University, Jinan, China

**Keywords:** LGI1 encephalitis, DNA methylation, Extracellular vesicles, Cytokines, Immune checkpoint molecular

## Abstract

**Background:**

Aberrant DNA methylation occurs commonly during pathogenesis of neuroimmunological diseases and is of clinical value in various encephalitis subtypes. However, knowledge of the impact of DNA methylation changes on pathogenesis of leucine-rich glioma-inactivated 1 (LGI1) antibody encephalitis remains limited.

**Methods:**

A total of 44 cytokines and 10 immune checkpoint moleculars (ICMs) in the serum of patients with LGI1 encephalitis and healthy donors (HDs) were measured to evaluate the association of them with clinical parameters. Genome-wide DNA methylation profiles were performed in peripheral blood mononuclear cell (PBMC) from LGI1 encephalitis patients and HDs using reduced representation bisulfite sequencing (RRBS) and validated for the methylation status by pyrosequencing. MicroRNA profiles were acquired in serum exosome by small RNA sequencing. Targeted cytokines expression was assessed at the presence or absence of miR-2467-5p in PBMCs and the culture media, and the binding of miR-2467-5p and its targeted genes was validated by luciferase assay.

**Results:**

There existed significant difference in 22 cytokines/chemokines and 6 ICMs between LGI1 encephalitis patients and HDs. Decreased PDCD1 with increased ICAM1 could predict unfavorable prognosis in one-year follow-up for LGI1 encephalitis patients. Fifteen of cytokines/chemokines and ICMs presented DNA-methylated changes in the promoter and gene body using RRBS in which five were verified as methylation status by pyrosequencing, and the methylation level of CSF3, CCL2, and ICAM1 was conversely associated with their expression in PBMCs. By combining RRBS data with exosome-derived microRNA sequencing, we found that hypomethylated-driven hsa-miR-2467-5p presented elevated expression in serum exosomes and PBMCs in LGI1 encephalitis. Mechanically, miR-2467-5p significantly induced reduced expression of CSF3 and PDCD1 by binding with their 3`UTR while enhanced CCL15 expression, but not significantly correlated with peripheral blood CD19 + B cell proportion of LGI1 encephalitis patients.

**Conclusions:**

Our results provided convincing evidence for DNA methylation changes, microRNA profiles in serum exosome for LGI1 encephalitis, and we also identified several novel cytokines related to clinical features in which some represented epigenetic modification of methylated-driven pattern and microRNA modulation. Our study contributed to develop treatment for epigenetic pathogenesis in LGI1 encephalitis.

**Supplementary Information:**

The online version contains supplementary material available at 10.1186/s13148-023-01550-5.

## Introduction

Patients with limbic encephalitis (LE) and antibodies against LGI1 antibody are typically older men with severe anterograde amnesia, psychiatric symptoms, and seizures, as well as medial temporal lobe abnormalities on brain MRI, but, bafflingly, frequently without inflammatory signs in cerebrospinal fluid (CSF) routine analysis [1, 2]. Faciobrachial dystonic seizures (FBDSs) are typical and frequently share clinical characteristics with other focal seizures [3]. LGI1 is a neuronal protein that is expressed primarily in the hippocampus. It forms a trans-synaptic complex with its partners ADAM23 and ADAM22 between Kv1.1 potassium channels and AMPA receptors [4]. Human antibodies to LGI1 prevent these interactions from occurring [5]. Anti-LGI1 encephalitis is strongly associated with genetic risk factors, including human leukocyte antigen (HLA) alleles DRB1*07:01 and DRB1*04:02 haplotypes in Whites and Asians, and DRB1*03:01 in Chinese anti-LGI1 cases [6, 7]. A recent study revealed hypomethylation of S100A6 and S100A11 DNA in white blood cell samples from patients with autoimmune encephalitis (AE). It was found that soluble S100A6 protein dramatically enhanced B lymphocyte infiltration across the blood–brain barrier (BBB), largely mirroring the impact of AE serum [8]. These results imply that changes in DNA methylation may be associated with the onset and progression of AE and that aberrant promoter CpG methylation provides a selective mechanism for regulating lymphocyte function in AE. The identification of epigenetically modified genes influencing the genesis, progression, and prognosis of LGI1 encephalitis is not well understood at this time. Consequently, we attempted to unravel DNA methylation changes in LGI1 encephalitis by high-throughput next-generation sequencing (NGS) for analyzing the DNA methylome and identifying methylation driver events on a genome-wide scale.

Exosomes are membrane-bound nanostructure vesicles shed by endocytic pathways and have been shown to mediate multiple biological/cellular processes required for the pathogenesis of neuroinflammatory disorders, such as multiple sclerosis, neuromyelitis optica spectrum disorders (NMOSDs), autoimmune encephalomyelitis, and others [9–11]. Exosomes contain a broad array of biological constituents, including proteins, lipids, transcriptional factors, and a wide variety of RNA and DNA [12]. A broad range of cellular miRNAs are packaged and transported into exosomes from AE patients, such as N-methyl-D-aspartate receptor (NMDAR) encephalitis, and gamma-aminobutyric acid B receptor (GABABR) encephalitis [13]. Exosome-derived miR-140-5p, for instance, could distinguish between patients with NMDAR encephalitis and those suffering from viral encephalitis [14]. Yet, microRNAs in exosomes from patients with LGI1 encephalitis were not completely profiled, nor was the biological function of specific microRNAs regulated by DNA methylation in the pathogenesis of LGI1 encephalitis investigated.

In this study, we evaluated the methylation profiles of 2 million CpG sites across the entire genome. Employing RRBS technology, coding genes and noncoding regions are identified. We identified several genes and one exosome-microRNA driven by DNA methylation as potential targets for inflammatory immunoreaction and epigenetic therapy in LGI1 encephalitis by combining this technology with sequencing of exosome miRNAs and an array of cytokines/chemokines/ICMs. We further investigated the relationship of these identified genes with clinical features. In LGI1 encephalitis, our findings further indicated the potential impact of exosome-microRNA on cytokine expression and PBMC function.

## Methods and materials

### Subjects and samples

Twenty individuals with anti-LGI1 encephalitis were recruited and given samples for this study between May 2018 and June 2021. (Additional file 13: Table S1). Using cell-based assays, the Graus and Dalmau criteria [15] were utilized to confirm the diagnosis of anti-LGI1 encephalitis and the identification of anti-LGI1 antibodies in serum or CSF. The control group included ten HDs of similar age and gender. Patients were excluded if they had: (1) less than a year of follow-up; (2) antibodies to surface antigens and other antigens with high clinical relevance (e.g., NMDAR, AMPAR, CASPR2, GABAA/BR, DPPX, Glycine receptor, AQP4, MOG, GFAP); (3) definite or suspected central nervous infection; and (4) definite or suspected systemic immune disease, toxic-metabolic encephalopathy, brain tumor. QAlb was age-normalized (QAlb/Qlim) by dividing QAlb by the age-dependent upper limit (Qlim; 4 + age/15 × 10^3^) [16].

All patients were recruited from Qilu Hospital, Cheeloo College of Medicine, Shandong University, and Shandong First Medical University's First Affiliated Hospital. The initial modified Ranking Scale (mRS) and neoplasm status of all patients were recorded. Data on CSF routine, immune therapy, and blood lymphocyte subset of 6 LGI1 encephalitis patients on admission and HDs were also gathered. Each patient's mRS score at the one-year follow-up visit was used to estimate the outcome. A favorable outcome was characterized as having an mRS score of less than two, whereas a poor outcome had an mRS value more than two. Pleocytosis was described as having more than 5 leucocytes per liter of CSF. High titer antibodies had titers greater than 1:100, whereas low titers had titers less than 1:100. According to QAlb/Qlim > 1, the blood-CSF barrier was designated as positive, and vice versa. Brain MRI abnormalities in LGI1 encephalitis usually referred to brain lesion sites (seen on MRI T2/FLAIR) in the medial temporal lobe, hippocampus, basal ganglia, and brainstem [17]. The human participant studies were investigated and approved by the ethical committees of Qilu Hospital, Cheeloo College of Medicine, Shandong University, and The First Affiliated Hospital of Shandong First Medical University. Each patient or a relative acting as his or her legal representative signed an informed consent form.

### Sample collection and antibody detection

Patients' fasting blood samples were drawn the day following their admission during active disease, prior to the initiation of immunotherapy, and immediately frozen at -80℃ until analysis. HD fasting samples were processed similarly to patient samples. Simcere Inspection Company (Nanjing, China) was given the gathered samples for commercial testing to detect antibodies to neuronal surface antigens and classical onconeuronal antibodies. An indirect immunofluorescence test was used to detect the antibodies.

### PBMC isolation and culture

Each subject's venous blood was taken in EDTA collection tubes. PBMCs were isolated by density-gradient centrifugation with Ficoll-Paque Plus (TBD Science, Tianjin, China) and cultured in RPMI 1640 (Gibco, Thermo Fisher Scientific, USA) with 10% fetal calf serum (Gibco, Thermo Fisher Scientific, USA), 100 g/ml streptomycin and 100 IU/ml penicillin, and 5% CO_2_ at 37 °C.

### Isolation of apoptotic body (AB), microvesicles, and exosome and validation

The serum samples were centrifuged at 300 g for 10 min to eliminate cell debris. The supernatant was centrifuged at 3000 g for 30 min to pellet extracellular vesicles of size AB. To isolate microvesicles, the residual supernatant was centrifuged at 13,000 g for 40 min at 4 °C, followed by 120,000 g for 70 min at 4℃. After washing and resuspending the pellets in PBS, the pellet suspension was centrifuged for 70 min at 120,000 g and 4℃ to isolate the exosome. Other techniques, like transmission electron microscopy, have been used to validate this isolation process (TEM, Tecnai G2 Spirit Bio TWIN, FEI, USA). Nanoparticle-tracking analysis was used to examine the size and distribution of these EVs (NTA).

### Human inflammation array and immune checkpoint molecule array

The Human Inflammation Array 3 was used to detect CXCL13, CCL11, CCL24, G-CSF (CSF3), GM-CSF, TCA-3, and other cytokines/chemokines in serum (RayBiotech). Human Immune Checkpoint Molecule Array 1 (RayBiotech) was used to detect ten human ICMs in serum: CD80, CD86, PD-L1, ICOSL, CD276, CD28, CTLA-4, ICOS, PD-1, and PD-L2. The testing was done according to the manufacturer's specifications. Every sample was diluted twice before being cultured on the array slide. The matching antibodies would attach to cytokines/ICMs in these samples, and biotinylated antibodies would be used to identify the bound molecules. GenePix was used to visualize and detect signals using Cy3-equivalent dye-conjugated streptavidin (Molecular Devices, USA). Signal data were calculated and analyzed with the Q-Analyzer program by subtracting the estimated background. Predetermined concentrations of array-specific cytokines and ICM standards were provided to generate a standard curve for cytokines and ICM quantification. By comparing sample signals to the standard curve, the cytokine concentration of each sample was measured. Each cytokine and ICM concentration was measured twice.

### Reduced representation bisulfite sequencing (RRBS)

According to the manufacturer's recommendations, PBMC DNA was extracted using the QIAamp Fast DNA Tissue Kit (Qiagen, Dusseldorf, Germany). Using the NanoDrop 2000 and an A260/280 range of 1.8–2.0, the DNA concentration was determined. MspI was used to do bisulfite conversion on fragmented DNA material (NEB, USA). Before PCR enrichment, size selection, and purification, single-stranded DNA fragments were linked to adapters with the Accel-NGS Methyl-Seq DNA Library Kit (Swift, MI, USA). Last but not least, pair-end 250 bp sequencing was performed on an Illumina Hiseq 4000 platform housed within the LC-bio facility (Hangzhou, China).

### DNA methylation analysis

Cutadapt and in-house Perl scripts were utilized to eliminate reads including adapter contamination, low-quality bases, and unclear bases. The quality of the sequence was then validated using FastQC (http://www.bioinformatics.babraham.ac.uk/projects/fastqc/). Bismark was utilized to map quality-approved readings to the reference genome. Following alignment, SAMTool was used to deduplicate the readings. For each cytosine site (or guanine corresponding to cytosine on the opposite strand) in the reference genome sequence, the DNA methylation level was determined by dividing the number of reads supporting C (methylated) by the total number of reads (methylated and unmethylated) using per scripts developed in-house and MethPipe. DMRs were calculated with the R package-MethylKit with the default parameters (1000 bp slide windows, 500 bp overlap, *p* value < 0.05).

### Small RNA sequencing and data analysis

Exosome total RNA was extracted using the miRNeasy Mini Kit (Qiagen, Dusseldorf, Germany). The quality and purity of the extracted RNA were determined using the Agilent 2100 Bioanalyzer (Agilent Technologies). The miRNA sequencing library was obtained using the TruSeq Small RNA Sample Prep kits (Illumina, San Diego, CA, USA). Single-end sequencing (1 × 50 bp) was then performed using the Illumina Hiseq2500 at the LC-bio (Hangzhou, China). Finally, we profiled the expression of miRNAs in the library using Illumina Hiseq2500.

### Pyrosequencing methylation analysis

Jieluoxuan Bio-Tech completed the pyrosequencing of bisulfite-treated DNA (Shandong, China). Genomic DNA was extracted from PBMC samples of LGI1 encephalitis patients and HDs using a QIAamp DNA Mini Kit (Qiagen, Dusseldorf, Germany) according to the manufacturer's instructions. 500 ng of DNA was treated with bisulfite using the EpiTect Bisulfite Kit (Qiagen) to convert all unmethylated cytosine to uracil while preserving 5-methylcytosine and then eluted in 35 μL of DNase-free water. Using the Pyromark Assay Design 2.0 (Qiagen, Dusseldorf, Germany), the sequencing primers were designed (Additional file 16: Table S4). Oligo-synthesized sequencing primers were created (Sangon Biotech, Shanghai, China). Following the instructions provided by the manufacturer of the pyrosequencing instrument, PyroMark Q48, pyrosequencing was performed (Qiagen). A paired t-test was conducted to determine whether the differences between LGI1 encephalitis and the control were statistically significant.

### Cell transfection

Once the PBMC cells reached 60–70% confluence in 24-well plates, transfection was conducted at a density of 1.0 × 10^6^ cells per well. Before transfection, the entire medium was replaced with a serum-free medium thirty minutes beforehand. To transfect the cells, INTERFERin (PolyPlus-transfection) was employed.

### Dual luciferase reporter assay

HEK-293 T cells were co-transfected with pmirGLO vector containing the wild-type or mutant CSF3 3`UTR and microRNA mimics using Lipofectamine 2000 (Invitrogen) as well as PDCD1 3`UTR and microRNA mimics. This was performed by Jieluoxuan Bio-Tech. After 48 h, luciferase activity was determined using the Dual-Luciferase Reporter Assay Kit (Promega, Madison, WI, USA) according to the manufacturer’s instructions. Firefly luciferase activity was normalized against Renilla luciferase activity.

### RNA isolation, reverse transcription, and qRT-PCR

Using TRIzon Reagent, total RNA was isolated from cells and EVs (CWBio, China). SYBR Green Pro Taq HS Kit was used to evaluate the expression of microRNA and mRNA (Accurate Biology, Jinan, China). The reverse transcription of RNA to cDNA was followed by Evo M-MLV RT Premix for qPCR (Accurate Biology, Jinan, China). The GAPDH gene was utilized as a reference. As internal control and external reference for miRNA expression in cells and EVs, respectively, U6 and cel-miR-39-3p (GenePharma, Shanghai, China) were selected. Using the 2Ct technique, the relative expression of miR-2467-5p and other genes was determined. The sequences of mRNA primers are provided in Additional file 17: Table S5.

### Enzyme-linked immunosorbent assays (ELISAs) for CSF3, PDCD1, and CCL15

The supernatants of cell cultures were collected and frozen in centrifuge tubes at -20℃. ELISA kits were used to measure CSF3, PDCD1, and CCL15 according to the manufacturer's instructions (Boster, Wuhan, China).

### Western blotting analysis

The samples were lysed in RIPA buffer (P0013B, Beyotime, China) with protease and phosphatase inhibitor cocktails added (TargetMol, USA). Through SDS-PAGE, total protein was isolated and transferred to PVDF membranes (Merck Millipore, Darmstadt, Germany). Following an hour of blocking in 5% nonfat milk, the membranes were incubated overnight at 4℃ with the following primary antibodies: anti-CD63 (1:2000, Abcam), anti-TGS101 (1:1000, Abcam), anti-ARF6 (1:1000, Cell Signaling Tech), and antibodies against C1QC (1:1000, Biosis, Beijing, China) and C3B (1:1000, Biosis, Beijing). After washing, membranes were incubated for one hour at room temperature with HRP-conjugated secondary antibodies (1:2000, Cell Signaling Technology). Using an Immobilon Western Chemiluminescent HRP substrate, signals were detected (Millipore, Darmstadt, Germany).

### Bioinformatics analysis

Using Targetscan (http://targetscan.org) and miRanda (http://microrna.org), we found the anticipated miRNAs targeting CSF3/PDCD1. Using the Metascape bioinformatics application (http://metascape.org), we analyzed Gene Ontology (GO) and KEGG pathways to identify the likely functions of enriched genes. Significant phrases were those with a P-value < 0.05, a minimum count of 3, and an enrichment factor > 1.5. To determine further the association between phrases, a selection of enriched terms was selected and shown as a network plot, with edges connecting terms with similarity of > 0.30. Using the databases BioGrid, InWeb IM, and OmniPath, an enrichment study of protein–protein interactions was done. Additionally, the Molecular Complex Detection (MCODE) algorithm was used to discover densely interconnected network components. Using topology analysis, the connectivity of the PPI network's nodes was analyzed to identify a greater proportion of essential nodes. The XIANTAO platform (www.xiantao.love) was utilized to analyze the connection between PDCD1 and CSF3 expression in thymoma and normal tissues from the TCGA database and their clinicopathologic characteristics. The XIANTAO platform assessed patient survival profiles based on the expression levels of PDCD1 and CSF3. Patients were stratified according on gender, and subsequently survival profiles were studied. The cutoff value for PDCD1/CSF3 expression to divide patients into high and low expression subgroups was calculated using the previously published minimal p-value method [18]. As indicated, the hazard ratio and log-rank p-value for each comparison were determined. On the XIANTAO platform, the immune infiltration profiles of tumors were evaluated. Twenty-four immunological markers were employed to differentiate between immunocytes. Using single-sample GSEA (ssGSEA), the Spearman correlations of immunocyte markers with PDCD1 and CSF3 expression levels were assessed.

### Statistical analysis

SPSS 22.0 was utilized for statistical analysis, and GraphPad Prism 8.0 was utilized to the figures (GraphPad Software, La Jolla, CA, USA). Continuous variables were represented by means standard deviations or medians with ranges. The χ^2^ or Fisher's exact test was utilized to compare discrete variables, and the Student's t test or the Mann–Whitney U test was utilized to evaluate quantitative data. One-way analysis of variance (ANOVA) was utilized to compare data between several groups. Spearman correlation was used to investigate parameter correlations. The area under the ROC curve (AUC) was calculated to evaluate the diagnostic performance of selected markers. ROC characterized the predictive function for distinguishing patients with encephalitis from controls, and AUC was used to evaluate the diagnostic performance of selected markers. *p* < 0.05 was used as the significance threshold for all comparisons.

## Results

### Demographic and clinical features of the patients with LGI1 encephalitis

Additional file 13: Table S1 presents the demographic information for 20 patients with LGI1 encephalitis and 10 HDs. The median age of patients with LGI1 encephalitis was 63.5 (55.0–69.0), and 40% were female. The median age of HDs was 54.45 (43.75–68.00) years old, and fifty percent of them were male. There was no difference between patients and HDs in terms of age or gender. Additional file 13: Table S1 also describes the clinical manifestations, CSF abnormalities, brain MRI, and treatment protocol in detail.

### Comparison of serum cytokines, chemokines, and ICMs between the patients with LGI1 encephalitis and HDs

**Fig. 1:**
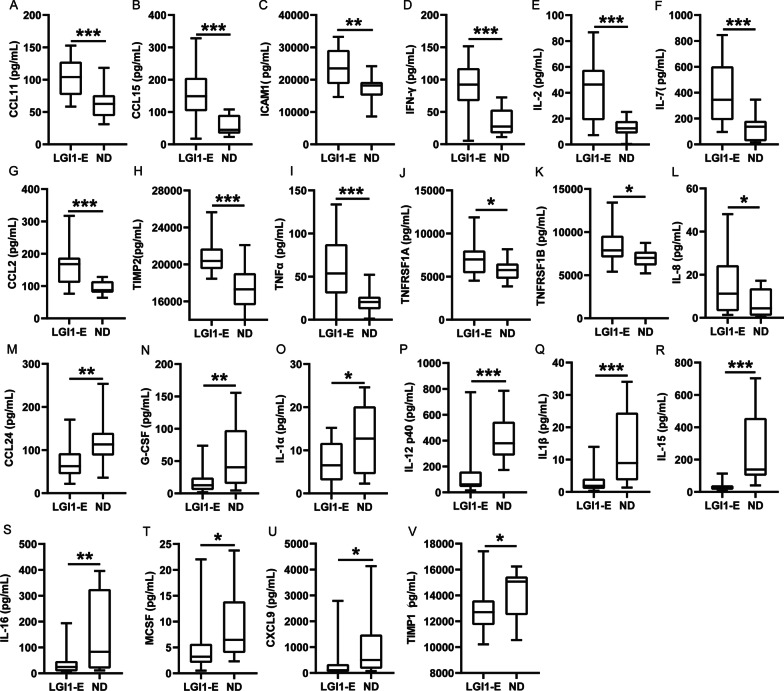
Cytokine/chemokine levels in serum compared between the patients with anti-LGI1 encephalitis and normal donors. **A**–**V** There were significant differences in serum level of CCL11, ICAM-1, CCL15, IFNγ, IL2, IL7, CCL2, TIMP2, TNFɑ, TNFRSF1A, TNFRSF1B, IL8, CCL24, G-CSF, IL-1 alpha (IL-1 F1), IL-12, IL-1 beta (IL-1 F2), IL-15, IL-16, M-CSF, CXCL9 and TIMP1 between LGI1 encephalitis cohort and control group. *LGI1-E* leucine-rich glioma inactivated-1 encephalitis; *ND* normal donor. *p < 0.05, **p < 0.01, ***p < 0.001

**Fig. 2:**
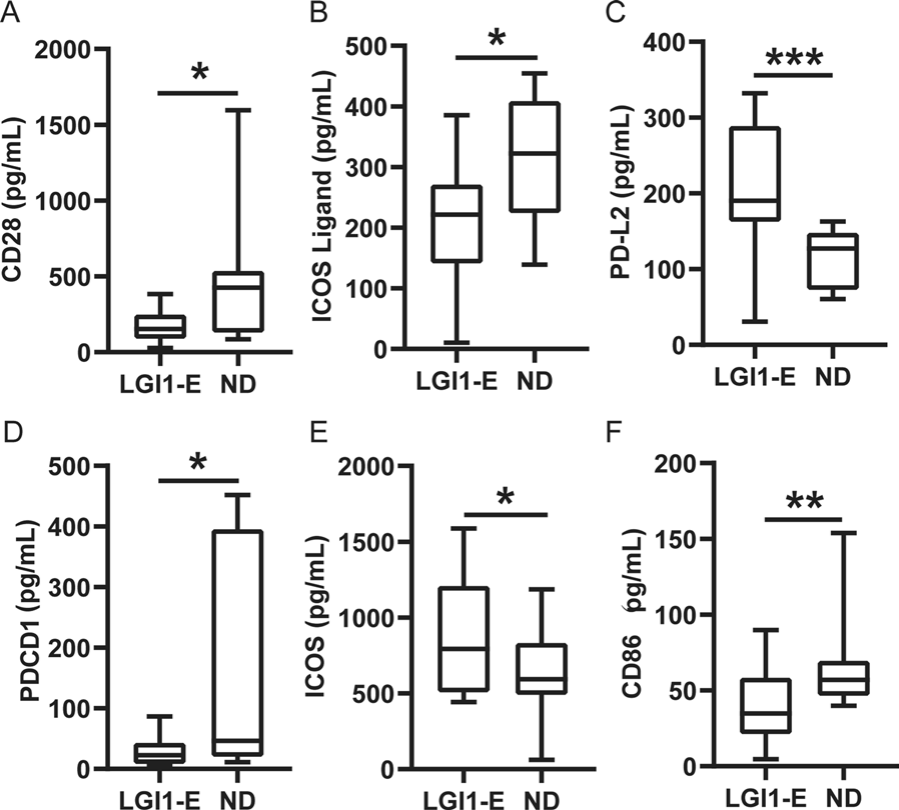
Immune checkpoint molecular levels in serum between the patients with LGI1 encephalitis and normal donors. **A**–**F** There were significant differences in serum level of CD28, ICOSL, PD-L2, PD1, ICOS and CD86 between anti-LGI1 encephalitis and normal donors. *LGI1-E* anti-leucine-rich glioma inactivated-1 encephalitis; *ND* normal donor. *p < 0.05, **p < 0.01, ***p < 0.001

In order to figure out the inflammatory factor profile of LGI1 encephalitis, cytokines antibody array was performed (Fig. 1), uncovering that the levels of CCL11 (*p* < 0.001), ICAM-1 (*p* < 0.01), CCL15 (*p* < 0.001), IFN-γ (*p* < 0.001), IL-2 (*p* < 0.001), IL-7 (*p* < 0.001), CCL2 (*p* < 0.001), TIMP2 (*p* < 0.001), TNFɑ (*p* < 0.001), TNFRSF1A (*p* < 0.05), TNFRSF1B (< 0.05) and IL-8 (*p* < 0.05) in serum were significantly increased in patients with LGI1 encephalitis compared to HDs. In contrast, the levels of CCL24 (*p* < 0.01), G-CSF (CSF3) (*p* < 0.01), IL-1 alpha (IL-1 F1) (*p* < 0.05), IL-12 p40 (*p* < 0.001), IL-1 beta (IL-1 F2) (*p* < 0.001), IL-15 (*p* < 0.001), IL-16 (*p* < 0.01), M-CSF (*p* < 0.05), CXCL9 (*p* < 0.05) and TIMP1 (*p* < 0.05) in serum were significantly decreased in patients with LGI1 encephalitis. As shown in Fig. 2, the serum of patients with LGI1 encephalitis had reduced levels of CD28, ICOS-L, PDCD1 (all *p* < 0.05), and CD86 (*p* < 0.01), but higher levels of ICOS (*p* < 0.05) and PD-L2 (*p* < 0.001) were detected. We examined these genes with differential expression and their PPI network via GO and pathway analysis (Additional file 1: Fig. S1). The levels of other cytokines/chemokines and ICMs were not different between LGI1 encephalitis patients and HDs (Additional file 2: Fig. S2). In addition, these 20 patients with LGI1 encephalitis were classified into three subgroups: 6 patients with anti-LGI1 antibody only in serum, 3 with anti-LGI1 antibody only in CSF, and 11 patients with anti-LGI1 antibody in serum and CSF. The CD28 expression in serum was significantly elevated in the patients with anti-LGI1 antibody only in serum, compared with the patients with anti-LGI1 antibody in serum and CSF and the patients with anti-LGI1 antibody only in CSF (*p* < 0.05). However, there was no differential expression for the remaining cytokines, chemokines, and immune checkpoint moleculars between these three subgroups (data not shown).

### Correlation between clinical features and cytokines, chemokines, and ICMs in patients with LGI1 encephalitis

**Fig. 3:**
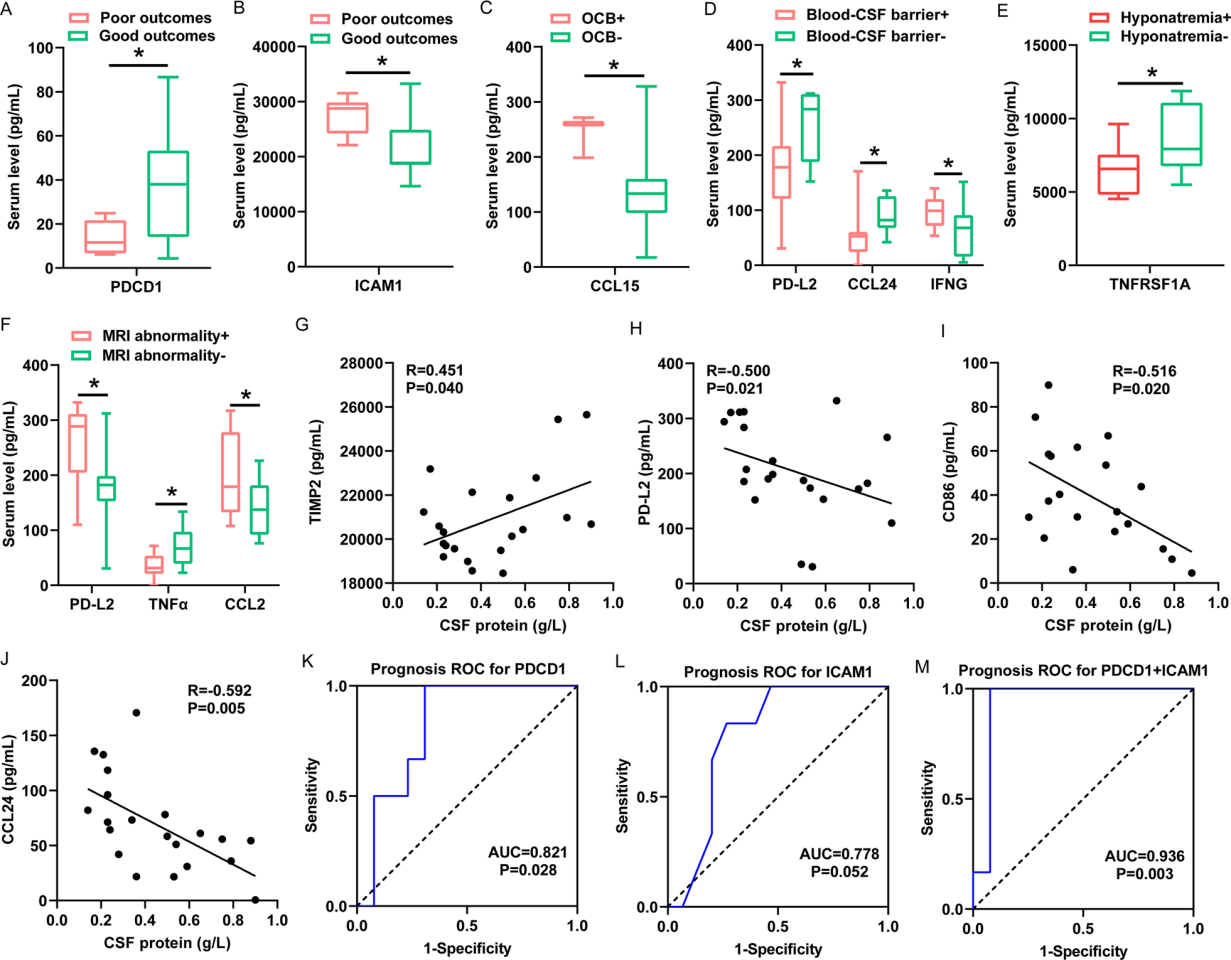
The association of cytokines/chemokines and immune checkpoint molecular in serum with clinical parameters. **A** and **B** The significant difference in serum level of PDCD1 and ICAM1 between LGI1 encephalitis patients with favorable and those with poor prognosis in one-year follow-up. **C** There were significant relationship between CCL15 and OCB occurrence in patients with LGI1 encephalitis. **D** The significant difference of serum PD-L2, CCL24 and IFNγ observed in LGI1 encephalitis patients with/without blood–CSF barrier dysfunction. **E** There was significant relationship between serum TNFRSF1A and hyponatremia in patients with LGI1 encephalitis. **F** The difference of serum PD-L2, TNFɑ, and CCL2 in LGI1 encephalitis patients with/without MRI abnormality. **G**–**J** The correlations between serum TIMP2, PD-L2, CD86, CCL24 and CSF protein level in the patients with LGI1 encephalitis. **K**–**M** ROC of PDCD1, ICAM1 and their combination for poor outcomes in one-year follow-up in LGI1 encephalitis patients. *CSF* cerebrospinal fluid. *p < 0.05

In these differentially expressed cytokines, chemokines, and ICMs as shown in Figs. 1 and 2, the serum level of PDCD1 tended to be lower in LGI1 encephalitis patients with poor outcomes than those with favorable outcomes (*p* < 0.05) (Fig. 3A), whereas ICAM1 level appeared significantly higher in LGI1 encephalitis patients with poor outcomes (*p* < 0.05) (Fig. 3B). The patients with LGI1 encephalitis with CSF-restricted OCBs exhibited higher level of CCL15 alone compared to those patients without CSF OCBs (*p* < 0.05) (Fig. 3C). In LGI1 encephalitis patients with blood–CSF barrier break, serum levels of IFN-γ were higher than those without barrier break, while serum levels of PD-L2 and CCL24 fell dramatically (all *p* < 0.05) in patients with blood–CSF barrier break (Fig. 3D). Hyponatremia was usually more frequent in LGI1 encephalitis patients with lower serum TNFRSF1A levels (*p* < 0.05) (Fig. 3E). This phenomenon was not observed in the rest differentially expressed factors. In addition, LGI1 encephalitis patients with brain MRI abnormalities presented the elevated level of PDL2 and CCL2 but lower levels of TNFα (all *p* < 0.05) in serum (Fig. 3F). We further investigated the correlation of all differentially expressed cytokines, chemokines, and ICMs with CSF protein level in LGI1 encephalitis. Our findings unraveled that a significant positive correlation existed between CSF protein level and serum TIMP2 expression (Fig. 3G), while CSF protein level was negatively associated with PD-L2, CD86, and CCL24 expression, respectively (Fig. 3H–J). The ROC curve also indicated that the combination of PDCD1 and ICAM1 could predict unfavorable prognosis of LGI1 encephalitis patients at one-year follow-up more accurately than either PDCD1 or ICAM1 alone (AUC = 0.936 *p* = 0.003 vs. AUC = 0.821 *p* = 0.028; AUC = 0.778 *p* = 0.052) (Fig. 3K–M).

### Overview of differentially methylated regions (DMRs) in PBMCs isolated from the patients with LGI1 encephalitis and HDs

RRBS was performed to study DNA methylation abnormalities in LGI1 encephalitis. Collecting six PBMC samples from patients with LGI1 encephalitis and five PBMC samples from HDs. The bisulfite conversion rate was 99.73%, and approximately 33 million reads were uniquely matched to the reference DNA genome (Additional file 14: Table S2). The average Pearson's correlation coefficient between control samples was greater than 0.946 (Additional file 3: Fig. S3A), illustrating excellent reproducibility of RRBS. Compared to control samples, the correlation coefficient between patient samples or between patient samples against control samples was lower (Additional file 3: Fig. S3A), indicating significant genome-wide DNA methylation variations between LGI encephalitis samples. Circos plots were applied to show the distribution of methylated CpG, CHG, and CHH sites on the chromosomes of patients with LGI1 encephalitis (Additional file 3: Fig. S3B) and HDs (Additional file 3: Fig. S3C).

**Fig. 4:**
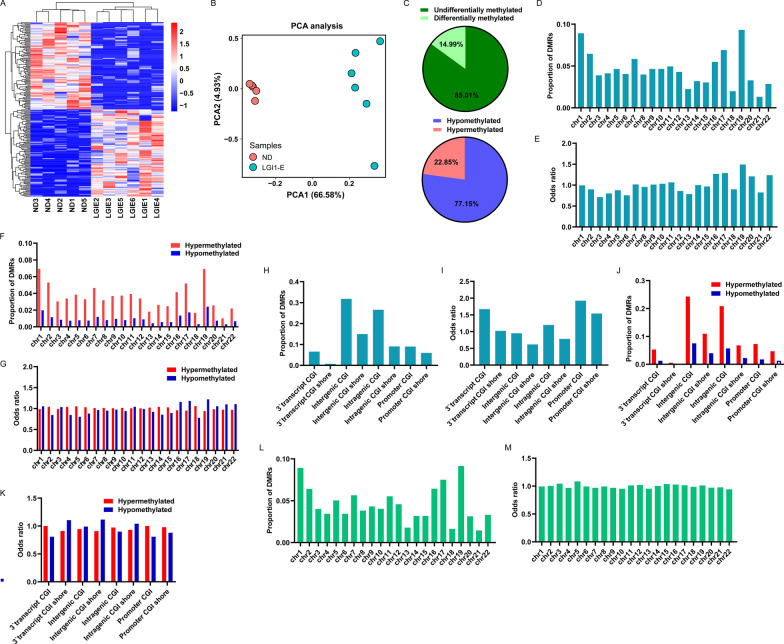
Characteristics of differentially methylated region in LGI1 encephalitis. **A** unsupervised hierarchical clustering based on the top 100 hyper-methylated and top 100 hypo-methylated CpG sites across 5 normal samples and 6 patients samples. Columns are samples and rows are CpG sites. **B** Principal component analysis of 5 normal samples and 6 patient samples based on methylation levels of differentially methylated CpG sites. **C** The proportion of all filtrated regions that are differentially methylated or not differential (up). The proportion of differentially hyper-methylated (red) or hypo-methylated (blue) regions (down). The proportion **D** and odds ratio **E** of differentially methylated regions (DMRs) in different chromosomes. The proportion **F** and odds ratio **G** of hyper-methylated (red) or hypo-methylated (blue) region in different chromosomes. The category **H** and odds ratio **I** of CGI locations for DMRs. The category **J** and odds ratio **K** of CGI locations for hyper-methylated (red) or hypo-methylated (blue) regions. The proportion **L** and odds ratio **M** of methylated regions correlated with the expression levels of genes in different chromosomes. Odds ratio was computed against the general distribution. DMRs, differential methylated regions; CGI, CpG island; shore, 0–2 kb from CpG island; promoter CGI, CGI located from 0 to 1000 bases upstream of the transcriptional start site (TSS) to 300 bases downstream of the TSS. Intragenic CGI, CGI located from 300th bases downstream of the TSS to 300th bases upstream of the transcriptional end site (TES); transcript CGI, CGI located from 300th bases upstream of the TES to 300th bases downstream of the TES; intergenic CGI, CGI located from 300th bases downstream of the TES to 1000th bases upstream of the TSS of next gene

Utilizing the top 100 upregulated and top 100 downregulated CpG sites across all data, unsupervised hierarchical clustering was conducted. Patient samples and normal samples were separated as expected (Fig. 4A). Principal component analysis (PCA) employing all differentially methylated CpG sites also demonstrated comparable segregation between these two groups (Fig. 4B). These results revealed substantial differences in methylation between the patients' PBMCs and the controls' PBMCs. We observed that within-patient variance was significantly greater than within-control variation (Fig. 4B), suggesting DNA methylation heterogeneity in PBMCs of LGI1 encephalitis patients.

When comparing patient samples with normal samples, 14.99% of regions were differentially methylated (Fig. 4C up), and 77.15% of these DMRs were hypo-methylated (Fig. 4C down). We conducted GO and pathway analyses on these genes with DMRs (Additional file 4: Fig. S4), presenting evidence that DNA-binding transcription factor activity was significantly enriched in GO function (Additional file 4: Fig. S4A). DMRs were predominantly enriched on Chromosome 19 (odds ratio (OR) = 1.49) and predominantly absent on Chromosome 3 (OR = 0.71). The majority of hyper-methylated sites were enriched on Chromosome 18 (OR = 1.06), while hypo-methylated regions were preferred on Chromosome 19 (OR = 1.22) (Fig. 4F, G). Moreover, DMRs were much more abundant in promoter CpG islands (CGI) than in other locations (Fig. 4H, I), with more hyper-methylated DMRs in promoter CGI than hypo-methylated DMRs (Fig. 4J, K). Hypo-methylated DMRs were likewise enriched in intergenic CGI shore (OR = 1.11), whereas hyper-methylated DMRs were also enriched in 3`transcript CGI (OR = 0.99) (Fig. 4J, K). Obviously, the methylation status of the promoter or gene-body DMRs corresponded considerably with the expression level of their host genes. These DMRs in the promoter or gene body were predominantly overrepresented on Chromosome 5 (OR = 1.08), according to our findings (Fig. 4L, M).

### Identification of novel methylation-driven genes in LGI1 encephalitis

**Fig. 5:**
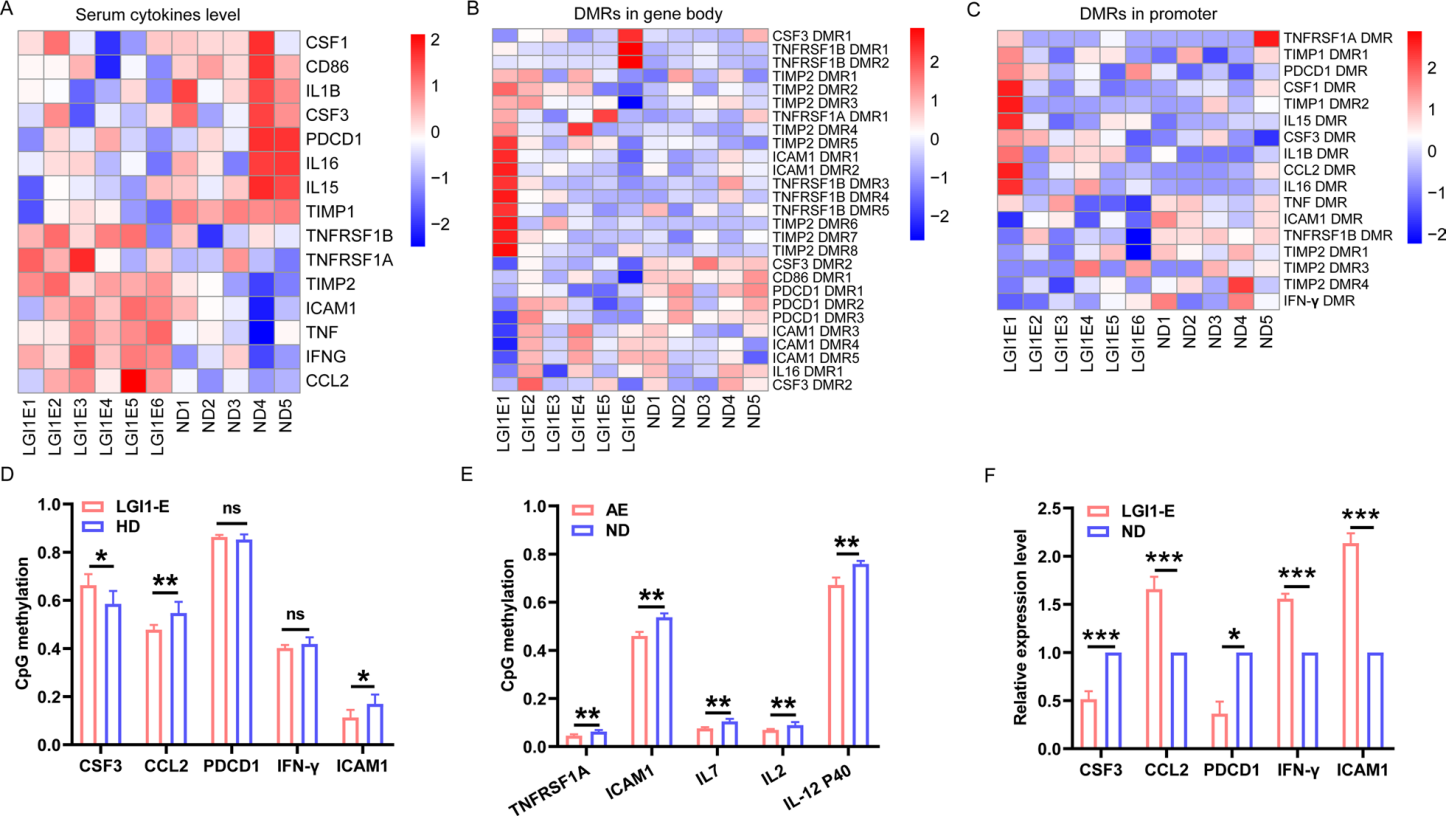
Methylation-driven cytokines/chemokines and ICMs in LGI1 encephalitis. **A** A heatmap representing discrepant serum protein expression of 15 methylation-driven cytokines/chemokines and ICMs in LGI1 encephalitis cohort compared with that in control group. **B** The multiple DMRs in gene body of 8 genes in LGI1 encephalitis cohort compared with that in control. **C** The methylation changes in promoter of 14 genes in LGI1 encephalitis cohort compared with normal donors. **D** Validation of methylation changes of 5 methylation-driven genes by bisulfite pyrosequencing using 5 encephalitis samples vs. 5 normal samples. **E** The external database GSE132866 showing differential DNA methylation level in promoter of TNFRSF1A, ICAM1, IL7, IL2 and IL-12 P40 in AE encephalitis cohort compared with normal donors. **F** PCR analysis of expression level of 5 gene in Fig. 5D between the patients with LGI1 encephalitis and normal donors. *LGI1-E* LGI1 encephalitis; *ND* normal donor; *AE* autoimmune encephalitis. Data are means ± SD of three experiments. *p < 0.05, **p < 0.01, ***p < 0.001

In our study, 15 of 28 differently expressed cytokines, chemokines, and ICMs showed methylated gene body or promoter modifications (Fig. 5A). Eight of these genes included multiple DMRs within the gene body (Fig. 5B), such as CSF3, TNFRSF1B, TIMP2 and ICAM1. Fourteen genes were found as having promoter methylation alterations (Fig. 5C). Using pyrosequencing, we confirmed the repeatability of the methylation alterations in the promoter of five novel genes (CSF3, CCL2, PDCD1, IFN-γ, and ICAM1) in another five PBMC samples from patients with LGI1 encephalitis and five control samples. Significant methylation alterations were found in the promoters of CSF3, CCL2, and ICAM1 in LGI1 encephalitis patients comparable to healthy individuals (Fig. 5D). In addition, our examination of the external data set GSE132866 demonstrated that hypomethylation in the promoters of TNFRSFA, ICAM1, IL-7, IL-2, and IL-12 P40 was more prevalent in AE patients than in HDs (Fig. 5E). As stated in our work, DNA methylation in the promoters of CSF3, PDCD1, IFN-γ, and CCL2 was not observed in GSE132866. This is most likely the result of sample heterogeneity in GSE 132866, where genomic DNA was extracted from blood white cells of patients with NMDAR encephalitis and unidentifiable AE. In addition, PBMCs were extracted from LGI1 encephalitis patients and HDs for PCR analysis of CSF3, CCL2, PDCD1, IFN-γ and ICAM1 expression levels. Our findings revealed an inverse relationship between CSF3 expression and its promoter methylation level as well as CCL2 and ICAM1. However, PDCD1 without methylation changes in promoter was significantly lower in PBMCs of LGI1 encephalitis patients compared with HDs. Similarly, IFN-γ displayed dramatically increased expression in PBMCs of LGI1 encephalitis patients but remained unchangeable in promoter methylation status (Fig. 5F). There was no association between methylation alterations in the promoters of these five genes and their expression in serum (Additional file 5: Fig. S5).

### The association of the methylation-silenced miR-2467-5p with expression level of CSF3, PDCD1, and CCL15 in PBMCs of patients with LGI1 encephalitis

**Fig. 6:**
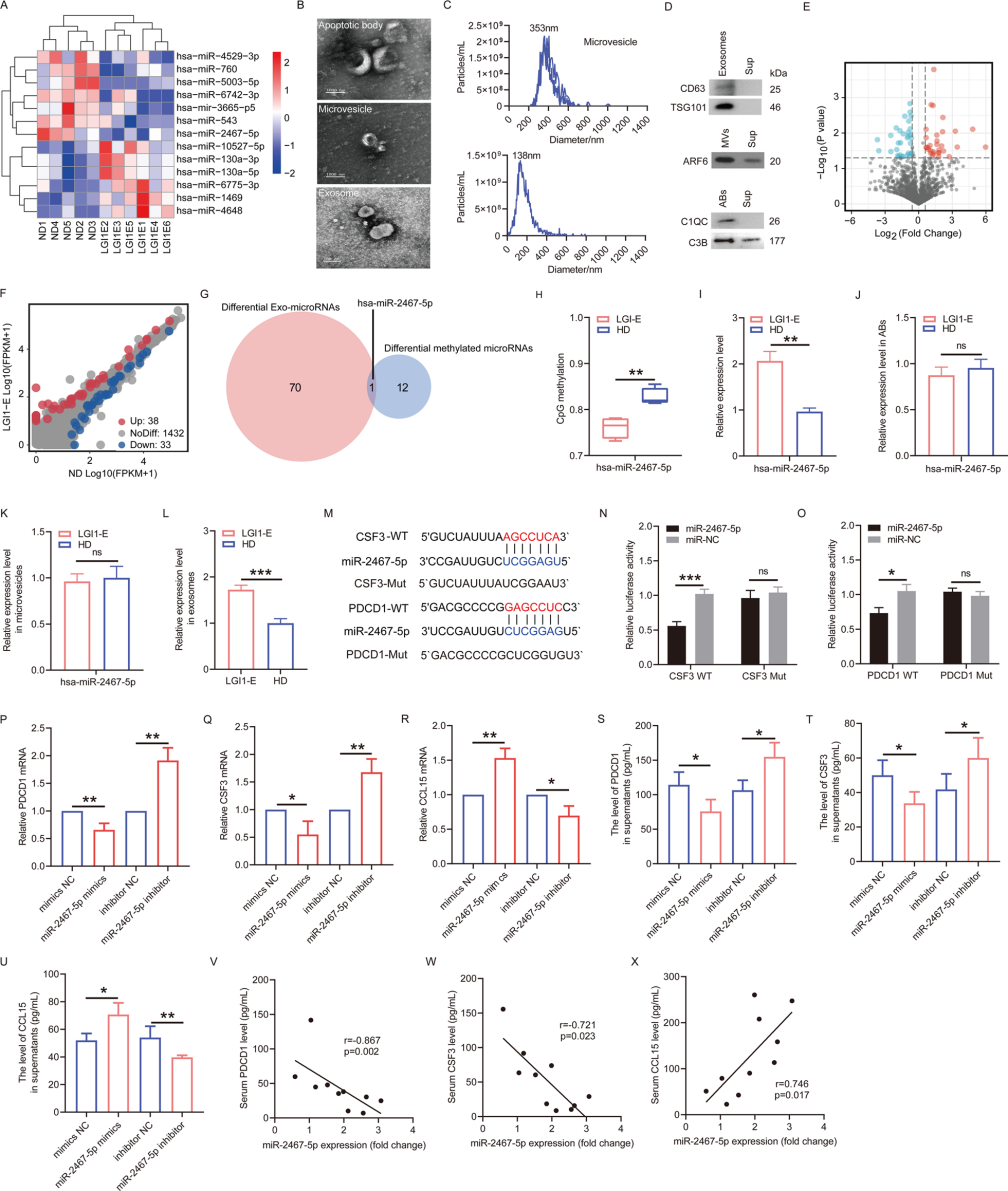
**A** A cluster heatmap showing methylation changes in promoter of 13 microRNAs between LGI1 encephalitis cohort and normal donors. **B** Morphological characteristics of apoptotic body, microvesicles, and exosome isolated from serum of one LGI1 encephalitis patient by TEM. **C** Particle size of microvesicles and exosome using NTA. **D** Protein markers of apoptotic body (C3B, C1QC), microvesicles (ARF6), exosome (CD63, TSG101) and serum supernatant by western blot. **E** Volcano plot of differentially expressive exosome-microRNAs between LGI1 encephalitis and healthy control. **F** A scatter plot assessing the expression variation of exosome microRNAs between LGI1 encephalitis patients and healthy donors. **G** Venn diagram showing the overlap of 71 differential exosome microRNAs and 13 methylated-driven microRNAs. **H** The methylation changes in promoter of hsa-miR-2467-5p between LGI1 encephalitis cases and healthy donors. **I** PCR analysis of miR-2467-5p expression in PBMCs between LGI1 encephalitis patients and healthy donors. **J** PCR analysis of miR-2467-5p expression in apoptotic bodies isolated from LGI1 encephalitis patients and healthy donors. **K** PCR analysis of miR-2467-5p in microvesicles isolated from LGI1 encephalitis patients and healthy donors. **L** PCR analysis of miR-2467-5p in exosomes isolated from LGI1 encephalitis patients and healthy donors. **M** Schematic representation of the complementary binding sites of CSF3 and PDCD1 with miR-2467-5p. Relative luciferase activity of wild-type and 3`UTR mutant constructs of CSF3 **N** and PDCD1 **O** cotransfected with miR-2467-5p mimics and miRNA-NC. PCR analysis of PDCD1 **P**, CSF3 **Q** and CCL15 **R** expression in PBMCs after transfection of miR-2467-5p mimics, inhibitor or scramble control into PBMCs. ELISA analysis of PDCD1 **S**, CSF3 **T,** and CCL15 **U** expression in cell supernatants after transfection of miR-2467-5p mimics, inhibitor or scramble control into PBMCs. Scatterplots showing the association of miR-2467-5p in PBMCs with the expression of PDCD1 **V**, CSF3 **W**, and CCL15 **X** in serum in LGI1 encephalitis patients (*n* = 6) and healthy donors (*n* = 4). *LGI1-E* LGI1 encephalitis; *ND* normal donor; *AE* autoimmune encephalitis; *MV* microvesicles; *ABs* apoptotic bodies; *Sup* supernatant; *NC* negative control. Data are means ± SD of three experiments. *p < 0.05, **p < 0.01, ***p < 0.001

RRBS data further indicated the considerably distinct methylation status of the promoters of thirteen microRNAs (Fig. 6A). To determine whether these 13 microRNAs were transferred into extracellular vesicles (EVs) in the circulation of patients with LGI encephalitis, we separated EVs into ABs (1000–5000 nm), microvesicles (100–1000 nm), and exosomes (30–150 nm) using differential centrifugation. Transmission electron microscopy was used to identify these particle classes and investigate their morphological properties (Fig. 6B). NTA was used to measure the size distribution of microvesicle and exosome (Fig. 6C). Anti-CD63 and anti-TSG101 for exosome markers, anti-ARF6 for microvesicles, and anti-C3B and anti-C1QC for apoptotic body were used for immunoblotting (Fig. 6D). We further investigated the size and concentration of exosomes and plasma particles between patients with LGI1 encephalitis and HDs. The size and concentration of exosomes did not change significantly between LGI1 encephalitis patients and HDs; however, the size of exosomes was frequently greater in LGI1 encephalitis patients than in HD patients (Figure S6A-S6E). Next, we examined the data on abundant exosome-derived microRNAs (exo-miRNAs) between LGI1 encephalitis cases and HDs (Additional file 15: Table S3). The volcano and scatter plots indicated the differences in exo-miRNAs between LGI1 encephalitis cases and HDs (Fig. 6E, F). The heating cluster map indicated 71 exo-miRNAs with differential expression between the LGI1 encephalitis cases and the control individuals (Additional file 6: Fig. S6F). A total of 38 exo-miRNAs with log_2_ fold change (FC) > 1 and *p* < 0.05 were deemed considerably upregulated, while 33 exo-miRNAs with log_2_FC < −1 and *p* < 0.05 were deemed significantly downregulated. Then, we conducted GO and pathway analyses to speculate on the potential functions of these exo-miRNAs (Additional file 6: Fig. S6G–L). The axon guidance pathway, for example, was more likely to be implicated in the pathological progression of LGI1 encephalitis since it was one of the KEGG pathways enriched for elevated microRNAs. Interestingly, methylation-driven has-miR-2467-5p was also identified as one of 71 exo-miRNAs with differential expression (Fig. 6G). Moreover, pyrosequencing analysis of has-miR-2467-5p demonstrated hypomethylated status in its promoter in LGI1 encephalitis patients comparable to HDs (Fig. 6H). Simultaneously, an increase in miR-2467-5p was observed in PBMCs and exosomes obtained from LGI1 encephalitis cases (Fig. 6I, L). Importantly, miR-2467-5p expression in ABs and microvesicles did not vary between LGI1 encephalitis cases and HDs (Fig. 6J, K). Next, the 436 targeted genes by miR-2467-5p were acquired through bioinformatic prediction of targetscan and miRDB (Additional file 7: Fig. S7A) and then were enriched for the potential biological processes (BP) and PPI network (Additional file 7: Fig. S7B–D). In addition, we investigated whether methylation alterations in the promoters of these 13 microRNAs affected their expression in exosomes from patients with LGI1 encephalitis and HDs (Additional file 8: Fig. S8). We found that increased DNA methylation level in the promoter of miR-2467-5p was not significantly correlated with its reduced expression in exosomes for LGI1 encephalitis cases and control participants (*r* = 0.393, *p* = 0.232) (Additional file 8: Fig. S8A). Intriguingly, DNA hyper-methylation status in the promoter of miR-543 was strongly associated with its elevated expression in exosomes for LGI1 encephalitis patients and HDs (Additional file 8: Fig. S8F). Then, bioinformatics algorithms uncovered the probable miR-2467-5p binding sites in the 3'untranslated region (UTR) of CSF3' and PDCD1' mRNA (Fig. 6M), and a negative correlation was found between PDCD1 expression and CCL15 expression in the serum of LGI1 encephalitis patients (r = −0.546, *p* = 0.013) (Additional file 9: Fig. S9). Therefore, we explored whether miR-2467-5p mediated the expression of CSF3, PDCD1, and CCL15 in PBMCs. The luciferase activity of wild type CSF3 3`UTR and PDCD1 3`UTR was evidently decreased in miR-2467-5p mimic group, but the luciferase activity of mutant CSF3 3`UTR and PDCD1 3`UTR remained unchanged after transfection of miR-2467-5p mimic (Fig. 6N, O). Then, PBMCs were isolated from the whole blood of four LGI1 encephalitis patients and cultured with miR-2467-5p mimics/inhibitors and scramble control. Then, cells and cell supernatant were used for PCR and ELISA analyses. As we found, miR-2467-5p overexpression led to a decrease in PDCD1 and CSF3 but an increase in CCL15 in the PBMCs of LGI1 encephalitis patients. In contrast, a reduction in miR-2467-5p significantly led to increased expression of CSF3 and PDCD1 and an decreased in CCL15 in the patients' PBMCs (Fig. 6P–R). A similar pattern was observed in the secretion of PDCD1, CSF3 and CCL15 (Fig. 6S–U). For LGI1 encephalitis patients (*n* = 6) and HDs (*n* = 4), miR-2467-5p expression in PBMCs was negatively associated with the serum levels of PDCD1 (*r* = −0.867, *p* = 0.002) and CSF3 (r = −0.721, *p* = 0.023), but favorably correlated with the serum level of CCL15 (*r* = 0.746, *p* = 0.017) (Fig. 6V–X).

### The association of miR-2467-5p with peripheral blood lymphocyte subsets in LGI1 encephalitis patients

**Fig. 7:**
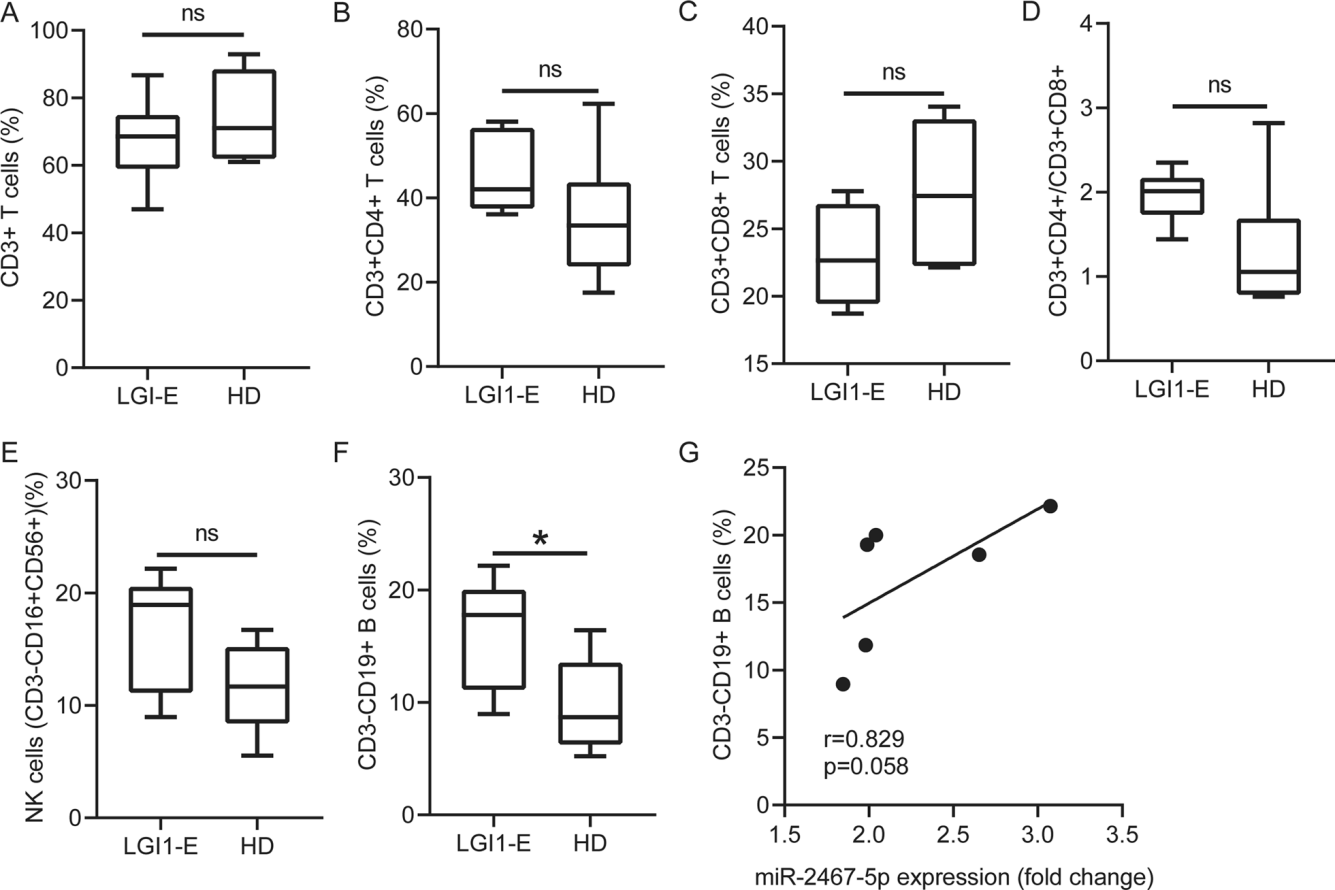
Peripheral blood lymphocyte subsets between LGI1 encephalitis cases and HDs. Flow cytometry analysis displayed the percentages of CD3 + T cells (**A**), CD3 + CD4 + T cells (**B**), CD3 + CD8 + T cells (**C**), the ratio of CD4 + T cells/CD8 + T cells (**D**), CD16 + CD56 + NK cells (**E**) and CD19 + B cells (**F**) in the peripheral blood of LG1 encephalitis patients (*n* = 6) compared with HDs (*n* = 6). **G** Correlation between miR-2467-5p expression in PBMCs and the proportion of peripheral blood CD19 + B cells in patients with LGI1 encephalitis (*n* = 6). *p < 0.05

We found that no significant difference existed in the proportion of the peripheral blood CD3 + T cells, CD3 + CD4 + T cells, CD3 + CD8 + T cells, the ratio of CD3 + CD4 + /CD3 + CD8 + and NK cells in LGI1 encephalitis patients (*n* = 6) compared with HDs (Fig. 7A–E). However, the percentage of CD19 + B cells was higher in the LGI1 encephalitis cases than in HDs (Fig. 7F). Positive correlation was not found between miR-2467-5p expression and the proportion of CD19 + B cells in the patients with LGI1 encephalitis (*r* = 0.829, *p* = 0.058) (Fig. 7G).

### The association of PDCD1 and CSF3 with human thymoma

**Fig. 8:**
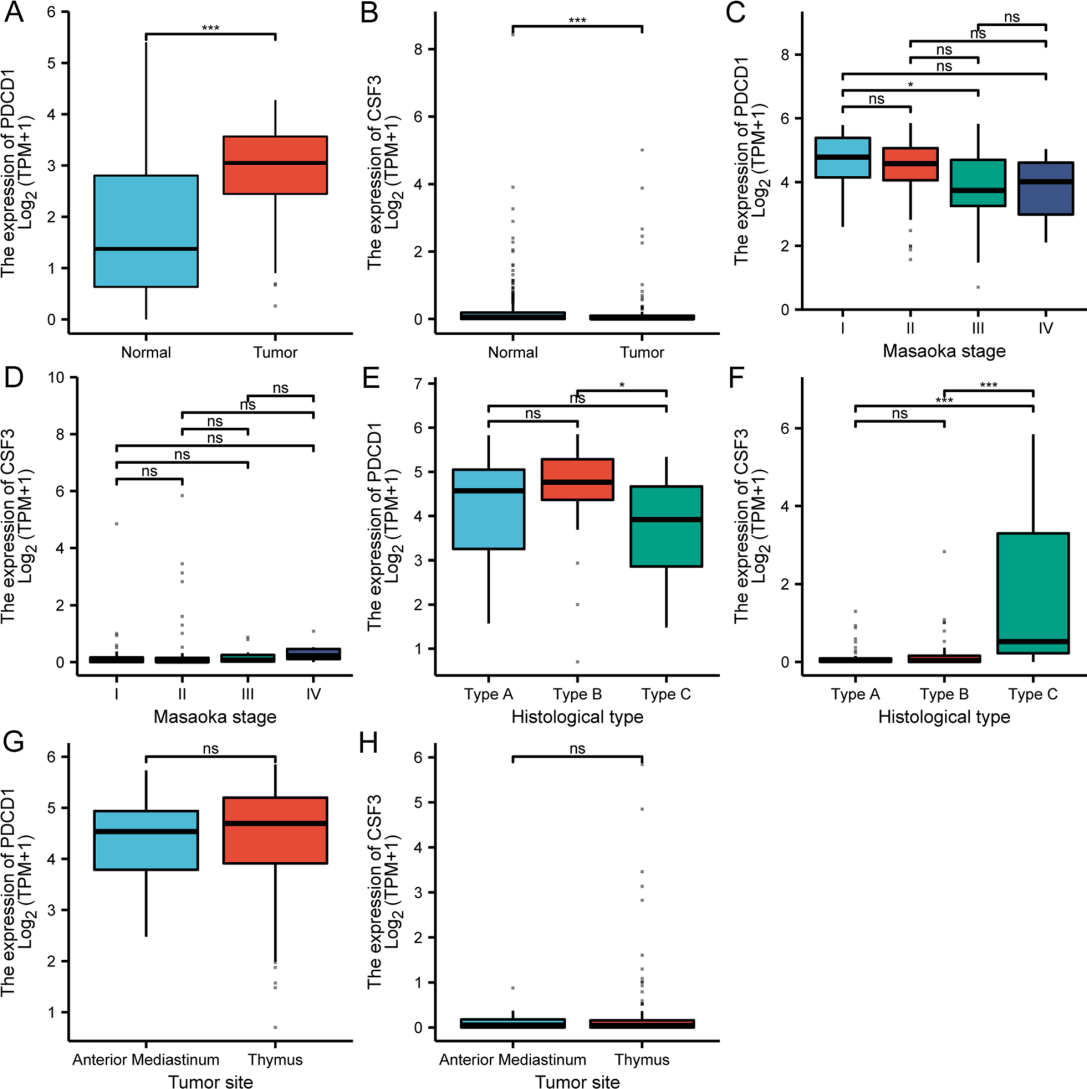
PDCD1 and CSF3 expression level in thymoma. **A** and **B** PDCD1 and CSF3 expression were compared between thymoma and normal tissues. **C** and **D** PDCD1 and CSF3 expression in thymoma were indicated in Masaoka stage I–IV. **E** and **F** PDCD1 and CSF3 expression in histological type A–C of thymoma. **G** and **H** PDCD1 and CSF3 expression in different tumor sites. All expression value (TPM value) were acquired using the TCGA dataset on the Xiantao platform. *ns* no significant. *p < 0.05, **p < 0.01, ***p < 0.001

Although LGI1 encephalitis is considered as non-paraneoplastic syndromes frequently [19], a minority of LGI1 encephalitis patients were diagnosed with a variety of tumor types, including thymoma, etc. [20]. We initially examined CSF3 and PDCD1 expression in diverse tumor tissue against normal tissue, revealing that PDCD1 expression was greater in the majority of tumor tissue versus unpaired/paired normal tissue (Additional file 10: Fig. S10A, C), whereas CSF3 expression was lower in the majority of tumor tissue (Additional file 10: Fig. S10B, D). PDCD1 expression was similarly greatly raised in thymoma, whereas CSF3 expression was significantly reduced (Fig. 8A, B). Based on the RNA sequencing data, PDCD1 expression was significantly lower in Stage-III thymoma than in Stage-I (Fig. 8C), although there was no difference in CSF3 expression between Masaoka stages I-IV (Fig. 8D). PDCD1 expression was found to be higher in histological type B compared to type C. (Fig. 8E). In contrast, CSF3 expression was considerably reduced in histological types A and B compared to type C (Fig. 8F). There was no difference in the expression of PDCD1 or CSF3 between the anterior mediastinum and the thymus (Fig. 8G, H). The clinical importance of PDCD1 and CSF3 expression in relation to patient survival and illness progression was then investigated. The impact of PDCD1 and CSF3 on patient survival was analyzed using the Kaplan–Meier plotting system. As indicated in Additional file 11: Figure S11A–C, the patients with higher PDCD1 expression and patients with higher CSF3 expression had non-significantly longer overall, disease-specific, and progression-free intervals (Additional file 11: Fig. S11D–F). Grouped by gender, the differential expression of PDCD1 and CSF3 did not alter patient survival, including overall, disease-specific, and progression-free interval survival (Additional file 12: Fig. S12).

**Fig. 9:**
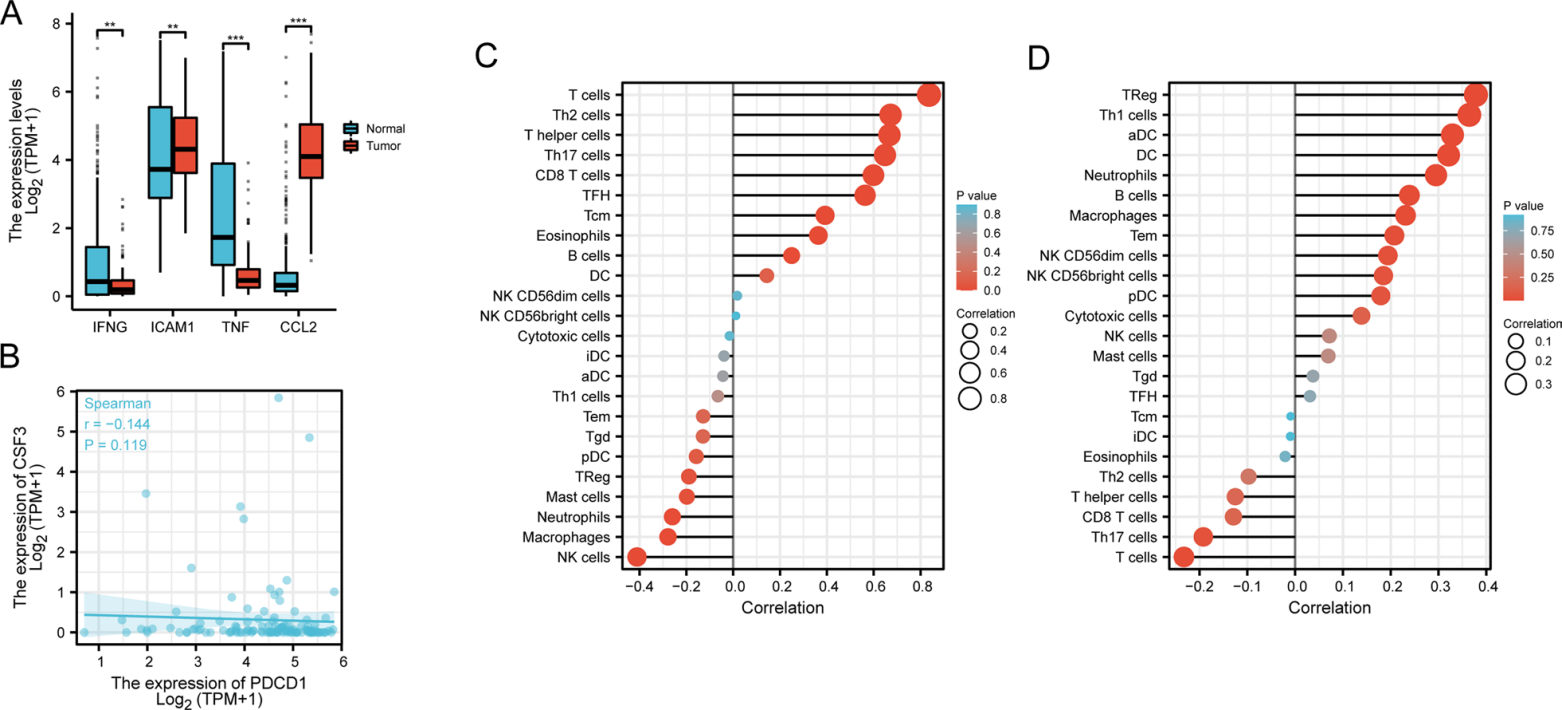
The association of PDCD1 and CSF3 expression with thymoma immune infiltration. **A** The expression of IFNγ, ICAM1, TNF and CCL2 were compared between thymoma and normal tissues using the TCGA dataset on the Xiantao platform. **B** Spearman correlation were shown between PDCD1 and CSF3 expression in thymoma. **C** and **D** The correlations of PDCD1 and CSF3 with all 24 immune cell types. *iDC* immature DC; *Tem* T effector memory; *TFH* T follicular helper; *Tgd* T gamma delta; *pDC* plasmacytoid DC; *aDC* activated DC; *Tcm* T central memory. *p < 0.05, **p < 0.01, ***p < 0.001

Based on the TCGA dataset, we also showed that IFN-γ, ICAM1, and TNF expression were decreased in thymoma; however, CCL2 expression was considerably increased (Fig. 9A). There was no significant association between CSF3 and PDCD1 in thymoma (Fig. 9B). Immune infiltration inside the tumor microenvironment is a significant element in determining anticancer efficacy and patient fate. We evaluated the effect of PDCD1 and CSF3 on thymoma immune infiltration patterns. Eight of the twenty-four types of invading immune cells exhibited a substantial positive correlate with PDCD1 expression (*r* > 0.30, *p* < 0.05) (Fig. 9C). Nonetheless, NK cells were correlated negatively with PDCD1 expression (*r* < −0.3, *p* < 0.05) (Fig. 9C). A significant positive association occurred between five types of immune cells and CSF3 expression (*r* > 0.30, *p* < 0.05) (Fig. 9D), including cytotoxic T cells, Th17 cells, neutrophils, and DC cells, all immune cells having a favorable correlation with PDCD1 and CSF3 showed anti-tumor properties.

## Discussion

Previous research has explored the profile of inflammatory markers in the serum/CSF of LGI1 encephalitis patients [21, 22]. However, these studies primarily examined various cytokines/chemokines in a small number of patient cases, and high-throughput screening for cytokines/chemokines based on protein array in LGI1 encephalitis, as well as ICMs, has not yet been described. Here, a thorough profile of inflammatory factors and ICMs in the serum of 20 patients with LGI1 encephalitis is compared to that of healthy controls. Notably, the serum concentrations of 12 inflammatory markers were significantly greater in the patients with LGI1 encephalitis, whereas the expression of the remaining 10 inflammatory factors was reduced in the patients. Similarly, ICOS and PD-L2 were found at a higher concentration in the serum of LGI1 encephalitis patients, whereas four ICMs were detected at a lower concentration compared to HDs.

In a recent study, the DNA methylation profiles of white blood cells from AE patients with NMDAR antibody and other nonspecific antibodies were investigated [8]. However, an NGS-based methylation examination of LGI1 encephalitis, particularly including these cytokines/chemokines and ICMs, has not yet been reported. RRBS was used to assess the genome-wide methylation patterns of PBMCs from the patients with LGI1 encephalitis. In the present study, on average, we were able to analyze methylation at over two million CpG sites per sample. Using the circular binary segmentation approach, we were able to identify 77.15% of DMRs with hypo-methylated status in our RRBS data, which could help us understand the role of DNA methylation in LGI1 encephalitis. As anticipated, we found extensive abnormalities of DNA methylation in LGI1 encephalitis, including global DNA hypomethylation and CGI-specific hypermethylation, indicating their crucial roles in the etiology of LGI1 encephalitis. Based on the results of the top two principal components of the PCA, we determined that the methylation patterns of PBMCs from the patients with LGI1 encephalitis were drastically distinct, which could partially explain patient heterogeneity. In contrast, their typical counterparts displayed comparable methylation landscapes. As shown in our findings, it is challenging to pinpoint driver events concerning inflammatory response and even T-cell immunomodulation from many DNA methylation changes in genome-wide methylation studies. In the present study, DNA methylation alterations in the gene body and promoters of 22 cytokines/chemokines and 6 ICMs with differential expression in LGI1 encephalitis patients and HDs were studied. Using this strategy, we identified 15 methylation-driven genes, 14 of which had hyper-or hypo-methylated promoters (e.g., TIMP1, CSF1, and IL15). ICAM1, CSF3, and CCL2 exhibiting methylation alterations, were validated in an independent cohort via pyrosequencing. In addition, our research revealed that the poor prognosis of LGI1 encephalitis patients was associated with an increase in ICMA1 expression. ICAM1 is a transmembrane protein whose expression is elevated on endothelial and epithelial cells at sites of inflammation, is expressed on leukocytes and endothelial cells, and is upregulated in response to inflammatory signals [23, 24]. ICAM1 has been linked to the pathophysiology of a number of autoimmune diseases and inflammatory syndromes, including gastric cancer, multiple sclerosis, and neuromyelitis optica [25–27]. ICAM-1-/- or mutant mice exhibited less T cell infiltration in the CNS and less clinical disability in a mouse model of multiple sclerosis [28]. In the anti-NMDAR encephalitis group, CSF levels of soluble ICAM1 were significantly higher in the patient cohort [23]. Notably, a significant positive association was observed between the severity of NMDAR encephalitis and the amount of ICAM1 in CSF [23]. The recruitment of circulating leukocytes to target tissues is an important stage in inflammatory diseases. A number of processes control leukocyte recruitment, including initial leukocyte binding, rolling along the blood vessel surface, and subsequent adhesion to the target. ICAM1 can mechanically assist circulating leukocytes in crossing the BBB and adhering to their target, as well as motivate the initiation and progression of auto-inflammatory responses, which have been implicated in the pathogenesis of NMDAR encephalitis [23] and may explain the poor prognosis of patients with LGI1 encephalitis with elevated ICAM1 levels in our study. We also showed that the MRI brain lesions were more prevalent in LGI1 encephalitis cases with a higher level of CCL2 than those with lower level of CCL2. CCL2, a pro-inflammatory chemokine, is responsible for inflammatory monocyte trafficking into the central nervous system through its interaction with C–C motif chemokine receptor type 2 [29]. The elevated level of CCL2 in brain suspension contributed to the increased permeability of BBB, hence enabling a greater infiltration of immune cells, including monocytes [30]. Anti-NMDAR antibody-induced recurrent seizures in mice were associated with an increase in CCL2 mRNA in the hippocampus [31]. In other seizure models, a similar pattern was observed [32, 33]. It was hypothesized that the distinct lesion in brain MRI of LGI1 encephalitis indirectly reflected the severity of lymphocyte transcellular migration across BBB and infiltration into CNS, which played a crucial role in initiating and developing the intracranial inflammatory reaction and subsequent cytotoxic effect on neuron and astrocyte injury.

Recently, epigenetic regulation of ncRNAs in AE has been characterized [13, 34]. Our findings revealed that the promoter of hsa-miR-2467-5p was present with hypo-methylation changes in LGI1 encephalitis for the first time. A prior study demonstrated that an increase in the NF-κB pathway induced miR-2467-5p in TNF-stimulated HeLa cells [35]. Similarly, we found that the level of serum TNFɑ was elevated in LGI1 encephalitis patients, concomitant with an increase in miR-2467-5p both in PBMCs and serum exosomes. Our in vitro experiment elucidated that higher expression of miR-2467-5p in PBMCs not only significantly induced silence of PDCD1 and CSF3 expression but enhanced CCL15 activation, and this phenomenon was also consistent with our findings that miR-2467-5p expression in PBMCs of LGI1 encephalitis patients was negatively correlated with the expression of PDCD1 and CSF3 in serum while positively correlated with CCL15 expression in serum. PDCD1 is a member of the immunoglobulin superfamily, mainly expressed in activated T cells, B cells, NK T cells, Treg cells, etc. [36]. The intracellular section of PDCD1 contains immunoreceptor tyrosine-based inhibitory motif (ITIM), recruiting SHP-2 and inhibiting downstream signaling molecules' phosphorylation, thus inhibiting lymphocyte proliferation and cytokine production [37]. PDCD1 knockout mice spontaneously developed lupus-like autoimmune symptoms, and immunoglobulin levels were significantly increased [38, 39]. The engagement of PDCD1 on T cells by either one of its ligands, PD-L1 or PD-L2 leads to the inhibition of T-cell proliferation [40]. PDCD1 and its two ligands, PD-L1 and PD-L2, have both membrane-bound and soluble forms [41]. It was reported that soluble PD-L1 and soluble PD-L2 could block the cell surface binding of PDCD1 to its functional ligand, thereby up-regulating immune response, increasing T cell proliferation in chronic infection, and participating in the anti-infection process [42]. Interestingly, our study revealed that higher soluble PD-L2 and lower PDCD1 were more frequent in the serum of LGI1 encephalitis patients compared to HD samples, which might facilitate peripheral T cell proliferation and T cell infiltrates in the white matter [43]. This might also explain our finding that the elevated soluble PD-L2 was also more frequent in the patients with brain MRI abnormality due to myelin breakdown and inflammatory cell infiltrates leading to localized edema in MRI scans [44]. Colony-stimulating factor 3 (CSF3) is one of several hematopoietic growth factors (HGFs) that control the production of circulating blood cells by the bone marrow [45]. Numbers of studies have all shown that CSF3 reduces systemic inflammatory activity by inhibiting the production or activity of the main inflammatory mediators interleukin-1 (IL-1), tumor necrosis factor-alpha (TNF-ɑ), and interferon-gamma (IFN-γ) [46, 47]. The decreased levels of CSF3 and other hematopoietic factors have been linked to Alzheimer’s disease (AD) and are, in fact, predictive of conversion from mild cognitive impairment (MCI) to AD [45]. In cerebral ischemia and neonatal hypoxia–ischemia models, CSF3 plays a neuroprotective function against BBB disruption and neuroinflammation to reduce infarct volume and improve neurological outcomes [48, 49]. In our study, a decrease in serum CSF3 was concomitant with an increase in TNFɑ and IFN-γ in serum. IFN-γ has been shown to decrease tight junction proteins in blood vessels, such as occludin [50], which was associated with BBB disruption in LGI1 encephalitis [43]. This was also line with our finding that the patients with BBB break usually had higher level of serum IFN-γ. LGI1 encephalitis was usually regarded as a non-paraneoplastic AE with a tumor incidence of 10% [51]; however, a minority of LGI1 encephalitis patients were affected by a range of tumors, including neuroendocrine pancreatic tumor, thymoma, abdominal mesothelioma, and others [1, 20]. According to Chinese expert consensus on the diagnosis and therapy of autoimmune encephalitis, the prevalent tumor subtype of LGI1 encephalitis in China is thymoma (2022 edition) [52]. Using the TCGA dataset, we focused on the expression pattern of PDCD1 and CSF3 in thymoma tissue. We found that PDCD1 expression was enhanced in the thymoma, but CSF3 expression decreased. The immune infiltration analysis of aberrant PDCD1 and CSF3 expression revealed that T cells, including Th2 cells, CD8 + T cells, and Th17 cells, were highly enriched and activated within the thymoma. The development of IgG4 antibodies is widely known to be T helper type 2 (Th2) and IL-4, IL-13, and IL-10-driven [53, 54], and the IgG subclass of LGI1 encephalitis antibody is definitely IgG4, implying that the enrichment of Th2 cells and Th17 cells in thymoma tissue was crucial for the production of harmful antibodies in LGI1 encephalitis. The detailed mechanism merits additional investigation. This research has some limitations. First, a small size of patient samples were recruited into our study, and hence, not all hyper/hypo-methylation-driven gene/microRNA were confirmed using pyrosequencing. Besides, a few samples used for flow cytometry analysis might contribute to the bias of the association between miR-2467-5p level in PBMCs with peripheral CD19 + B cell population, partly due to the sample heterogeneity, such as sampling time, sample storage, and test conditions. This also limited our findings of the association between lymphocyte subsets and the initiation and progression of LGI1 encephalitis. Second, the origin of miR-2467-5p in exosome was unclear and needed to be investigated in various lymphocyte subsets. Importantly, no well-accepted animal model of LGI1 encephalitis was developed until now, although a mouse model based on cerebroventricular transfer of patient-derived IgG was reported by Mar Petit-Pedrol and co-workers, but several issues remain unresolved [4]. Therefore, we did not validate in vivo the aberrant methylation changes of novel genes related to clinical features and investigate the impact of these genes on the dysfunction of immune reaction in LGI1 encephalitis (i.e., ICAM1, PDCD1,CSF3). The biology and functionality of targeted genes in differentiating T cell and B cell subtypes in LGI1 encephalitis are not addressed in here.

## Conclusion

Taken together, we uncovered a substantial number of hypermethylation/hypomethylation events pre-marked by the poised promoter and exosome-derived microRNA profiles in LGI1 encephalitis. By combining these sequencing data with cytokines/chemokines and ICMs array, our experiment validated that the expression of three cytokines and exosome-derived miR-2467-5p was conversely mediated by methylation status in promoter. Clinically, ICAM1 and PDCD1 could serve as prognostic predictors of LGI1 encephalitis at one-year follow-up. Mechanistically, exosome-derived miR-2467-5p led to aberrant expression of CSF3, PDCD1 and CCL15 in PBMCs. In all, our study provides a significant resource for future efforts to establish prognostic biomarkers based on DNA methylation and exosome microRNAs, and creates an epigenetic therapy for LGI1 encephalitis. 

### Supplementary Information

**Additional file 1**: **Fig**. **S1**. GO, KEGG and PPI network analysis of differential expressive cytokines/chemokines and Immune checkpoint molecular. Column plot showing biological process (**A**), molecular function (**B**) and cellular component (**C**) with the respective significantly enriched terms. (**D**) KEGG enriched cluster of differential expressed cytokines/chemokines and Immune checkpoint molecular via Metascape. (**E**) The genes in Fig.D were colored by their *P*-value. (**F**) PPI network presentation of differential expressed cytokines/chemokines and Immune checkpoint molecular using Metascape.

**Additional file 2**: **Fig**. **S2**. Serum cytokines/chemokines and immune checkpoint molecular without differential expression between the patients with LGI1 encephalitis and healthy donors. **A**–**P** The serum level of CCL3, CCL5, CCL15, CXCL13, TCA-3, IL-1 F3, IL-4, IL-5, IL-6, IL-6R, IL-10, IL-11, IL-12 p70, IL-13, IL-17A, PDGFB was found no difference between LGI1-E cases and normal donors. **Q**–**T** There was no difference in serum CD80, CD276, CTLA4 and PDL1 level between LGI1-E cases and normal donors. LGI1-E, LGI1 encephalitis; ns, no significance.

**Additional file 3**: **Fig**. **S3** (**A**) Box plots of Pearson correlation coefficients calculated among normal samples, LGI1 encephalitis samples, and normal vs LGI1 encephalitis, respectively. Circular plot of differentially methylated CpG, CHG and CHH sites for LGI1 encephalitis patients (**B**) and normal donors (**C**). ND, normal donor.

**Additional file 4**: **Fig**. **S4**. GO and KEGG enrichment analysis of genes with DMRs. Dot plot showing biological process, molecular function, cellular component (**A**) and KEGG (**B**) with the significantly enriched terms.

**Additional file 5**: **Fig**. **S5**. The association of the methylation changes of DMRs within the promoter of 6 novel methylation genes with their concentration in serum. Scatterplots and box plots showing the correlation of methylation level of DMR within the promoter of CSF3 (**A**), PDCD1 (**B**), IFN-γ (**C**), ICAM1 (**D**), TNFɑ (**E**) and CCL2 (**F**) with their expression level in serum.

**Additional file 6**: **Fig**. **S6**. The expression profiles of microRNAs in exosome and their potential functionality. NTA analysis the size of EVs (**A**), EV concentration (**B**), the concentration of particle within the diameter 50–150nm (**C**), particle size and concentration in plasma (**D** and **E**). (**F**) A heat map representing discrepant microRNA expression values in plasma exosome of LGI1 encephalitis patients compared with those of healthy control. (**G**) and (**H**) KEGG pathways for 38 significant upregulated exo-microRNAs and 33 downregulated exo-microRNAs. (**I**) and (**J**) BP enrichment for 38 significant upregulated exo-microRNAs and 33 downregulated exo-microRNAs. (**K**) and (**L**) MF enrichment for 38 significant upregulated exo-microRNAs and 33 downregulated exo-microRNAs. LGI1-E, LGI1 encephalitis; ND, normal donor.

**Additional file 7**: **Fig**. **S7**. The potential BP enrichment for exo-miR-2467-5p. (**A**) The 436 overlapped genes targeted by miR-2467-5p were acquired through bioinformatic tools. (**B**) The potential BP of 436 overlapped genes using Metascape. (**C**) The genes in Fig. S7B were colored by their match P value. (**D**) The enriched cluster showing the interaction of 10 modules in PPI network as analyzed by Metascape.

**Additional file 8**: **Fig**. **S8**. The association of the methylation status of DMRs within the promoter of 13 differentially methylated microRNAs with their expression in plasma exosome. Scatterplots and box plots showing methylation level of DMR within the promoter of miR-2467-5p (**A**), miR-4529-3p (**B**), miR-760 (**C**), miR-5003-5p (**D**), miR-3665-5p (**E**), miR-543 (**F**), miR-10527-5p (**G**), miR-130a-3p (**H**), miR0130a-5p (**I**), miR-6775-3p (**J**) and miR-1469 (**K**) and their matched expression in exosome between LGI1-E patients and normal donors. *LGI1-E* LGI1 encephalitis; *ND* normal donor.

**Additional file 9**: **Fig. S9**. The association of the level of plasma PDCD1 and CSF3 with multiple cytokines expression in plasma in LGI1 encephalitis patients. Scatterplots showing the association of PDCD1 level with the expression of CCL2 (**A**), CCL15 (**B**), CCL24 (**C**), ICAM1 (**D**), IFN-γ (**E**), PD-L2 (**F**) and TNF-ɑ (**G**) in LGI1 encephalitis. Scatterplots showing the association of CSF3 level in plasma with the expression of CCL2 (**H**), CCL15 (**I**), CCL24 (**J**), ICAM1 (**K**), IFN-γ (**L**), PD-L2 (**M**) and TNF-ɑ (**N**) in LGI1 encephalitis.

**Additional file 10**: **Fig. S10**. PDCD1 and CSF3 expression in human cancers. **A** PDCD1 expression profiles in human cancers were compared with normal tissues using the TCGA dataset on the XIANTAO platform. **B** CSF3 expression profiles in human cancers were compared with normal tissues using the TCGA dataset. **C** PDCD1 expression profiles in human cancers were compared with the paired tissues using the TCGA dataset. **D** CSF3 expression profiles in human cancers were compared with the paired tissues using the TCGA dataset. *P < 0.05, **P < 0.01, ***P < 0.001.

**Additional file 11**: **Fig. S11**. The correlates of PDCD1 and CSF3 expression with survival outcome in thymoma patients. **A**–**C** Kaplan-Meier analysis of the correlation of PDCD1 expression with overall survival, disease-specific survival and progress free interval in thymoma patients. **D**–**F** Kaplan–Meier analysis of the correlation of CSF3 expression with overall survival, disease-specific survival and progress free interval in thymoma patients.

**Additional file 12**: **Fig. S12**. The correlates of PDCD1 and CSF3 expression with survival outcome in female or male patients with thymoma. **A–C** Kaplan–Meier analysis of the correlation of PDCD1 expression with overall survival, disease-specific survival and progress free interval in female with thymoma. **D**–**F** The correlation of PDCD1 expression with overall survival, disease-specific survival and progress free interval in male with thymoma. **G**–**I** The correlation of CSF3 expression with overall survival, disease-specific survival and progress free interval in female with thymoma. **J**–**L** The correlation of PDCD1 expression with overall survival, disease-specific survival and progress free interval in male with thymoma. Case numbers in each group were listed at the bottom of the figure.

**Additional file 13**: The demographic and clinical features of the patients with anti-LGI1 encephalitis and healthy donors.

**Additional file 14**: Sequencing quality of RRBS.

**Additional file 15**: Sequencing quality of exosome miRNA-seq.

**Additional file 16**: A list of primers used for pyrosequencing validation.

**Additional file 17**: qPCR primers used in the vitro assays.

## Data Availability

All data used during the current study available from the corresponding author on reasonable request.

## Abbreviations

LGI1: Leucine-rich glioma-inactivated 1
ICMs: Immune checkpoint moleculars
HDs: Healthy donors
PBMC: Peripheral blood mononuclear cell
RRBS: Reduced representation bisulfite sequencing
LE: Limbic encephalitis
CSF: Cerebrospinal fluid
FDBS: Faciobrachial dystonic seizures
HLA: Human leukocyte antigen
AE: Autoimmune encephalitis
BBB: Blood–brain barrier
NGS: Next-generation sequencing
NMOSDs: Neuromyelitis optica spectrum disorders
NMDAR: N-methyl-D-aspartate receptor
GABABR: Gamma-aminobutyric acid B receptor
AB: Apoptoticbody
EVs: Extracellular vesicles
NTA: Nanoparticle-tracking analysis
ELISA: Enzyme-linked immunosorbent assay
GO: Gene Ontology
MCODE: Molecular Complex Detection
ssGSEA: Single-sample GSEA
ANOVA: One-way analysis of variance
AUC: The area under the ROC curve
DMRs: Differentially methylated regions
PCA: Principal component analysis
UTR: Untranslated region
CNS: Central nervous system
ncRNAs: Non-coding RNAs
ITIM: Immunoreceptor tyrosine-based inhibitory motif
CSF3: Colony-stimulating factor 3
HGFs: Hematopoietic growth factors
IL-1: Interleukin-1
TNF-α: Tumor necrosis factor-alpha
IFN-γ: Interferon-gamma
MCI: Mild cognitive impairment
Th2: T helper type 2
