# Supplementary figures and images for: Abnormal DNA methylation analysis of leucine-rich glioma-inactivated 1 antibody encephalitis reveals novel methylation-driven genes related to prognostic and clinical features
Source: Clin Epigenetics. 2023 Aug 29;15:139. doi: 10.1186/s13148-023-01550-5 (PMC10463459; doi:10.1186/s13148-023-01550-5)

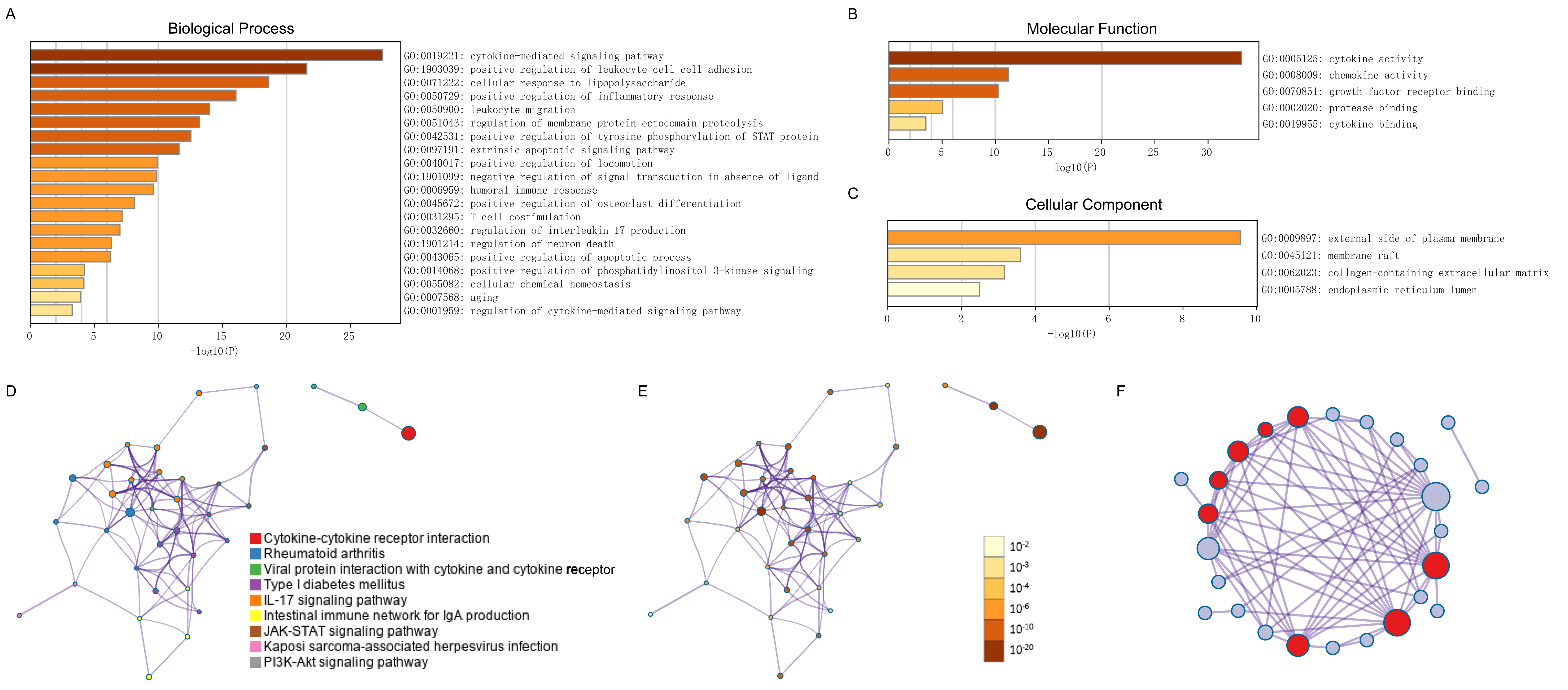

Supplement: Supplementary file 1 — Additional file 1: Fig. S1. GO, KEGG and PPI network analysis of differential expressive cytokines/chemokines and Immune checkpoint molecular. Column plot showing biological process (A), molecular function (B) and cellular component (C) with the respective significantly enriched terms. (D) KEGG enriched cluster of differential expressed cytokines/chemokines and Immune checkpoint molecular via Metascape. (E) The genes in Fig.D were colored by their P-value. (F) PPI network presentation of differential expressed cytokines/chemokines and Immune checkpoint molecular using Metascape. [file 13148_2023_1550_MOESM1_ESM.tif]

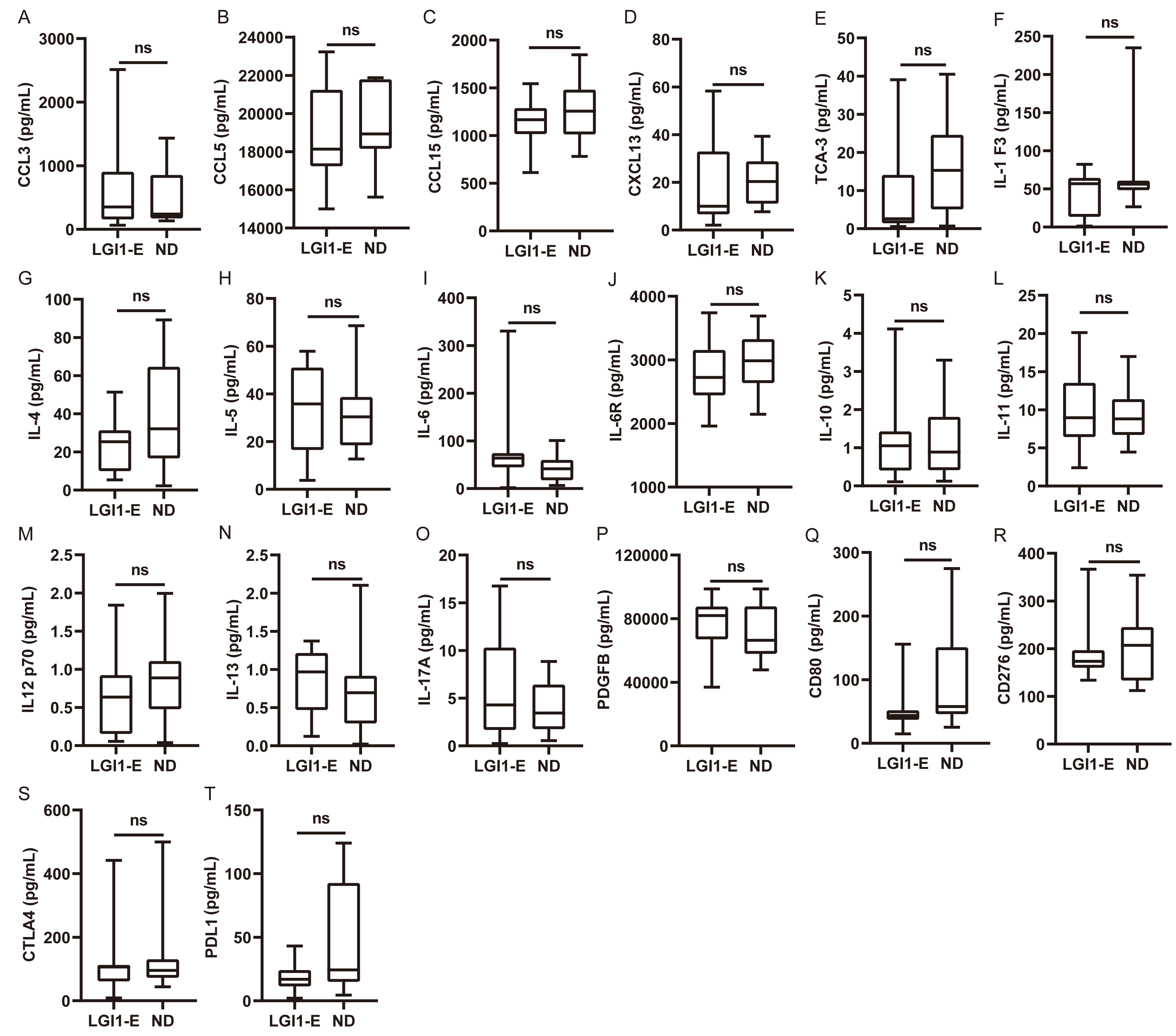

Supplement: Supplementary file 2 — Additional file 2: Fig. S2. Serum cytokines/chemokines and immune checkpoint molecular without differential expression between the patients with LGI1 encephalitis and healthy donors. A–P The serum level of CCL3, CCL5, CCL15, CXCL13, TCA-3, IL-1 F3, IL-4, IL-5, IL-6, IL-6R, IL-10, IL-11, IL-12 p70, IL-13, IL-17A, PDGFB was found no difference between LGI1-E cases and normal donors. Q–T There was no difference in serum CD80, CD276, CTLA4 and PDL1 level between LGI1-E cases and normal donors. LGI1-E, LGI1 encephalitis; ns, no significance. [file 13148_2023_1550_MOESM2_ESM.tif]

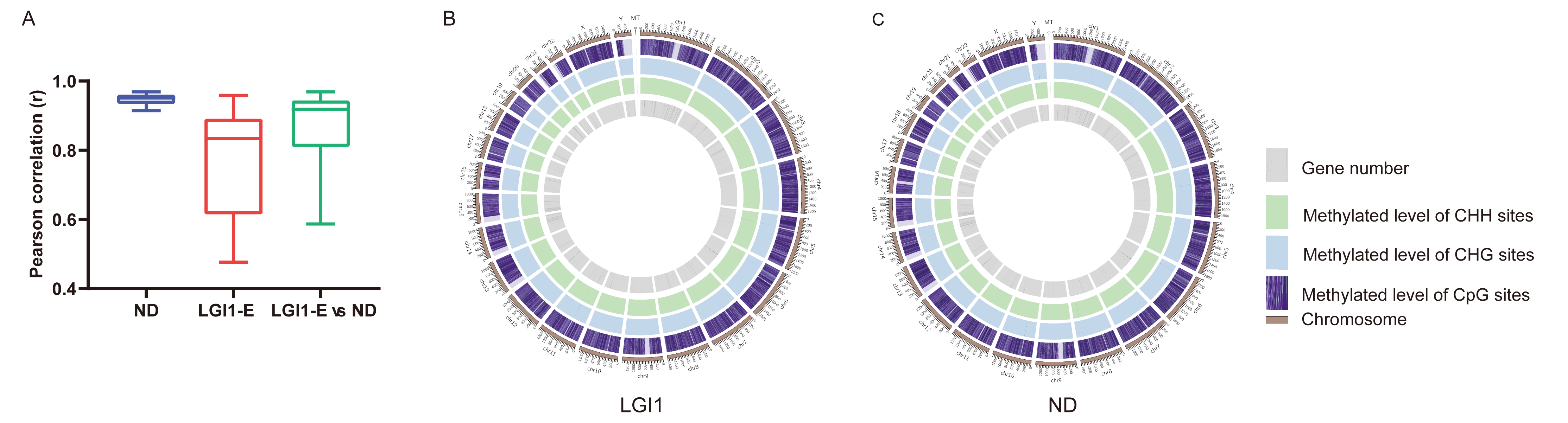

Supplement: Supplementary file 3 — Additional file 3: Fig. S3 (A) Box plots of Pearson correlation coefficients calculated among normal samples, LGI1 encephalitis samples, and normal vs LGI1 encephalitis, respectively. Circular plot of differentially methylated CpG, CHG and CHH sites for LGI1 encephalitis patients (B) and normal donors (C). ND, normal donor. [file 13148_2023_1550_MOESM3_ESM.tif]

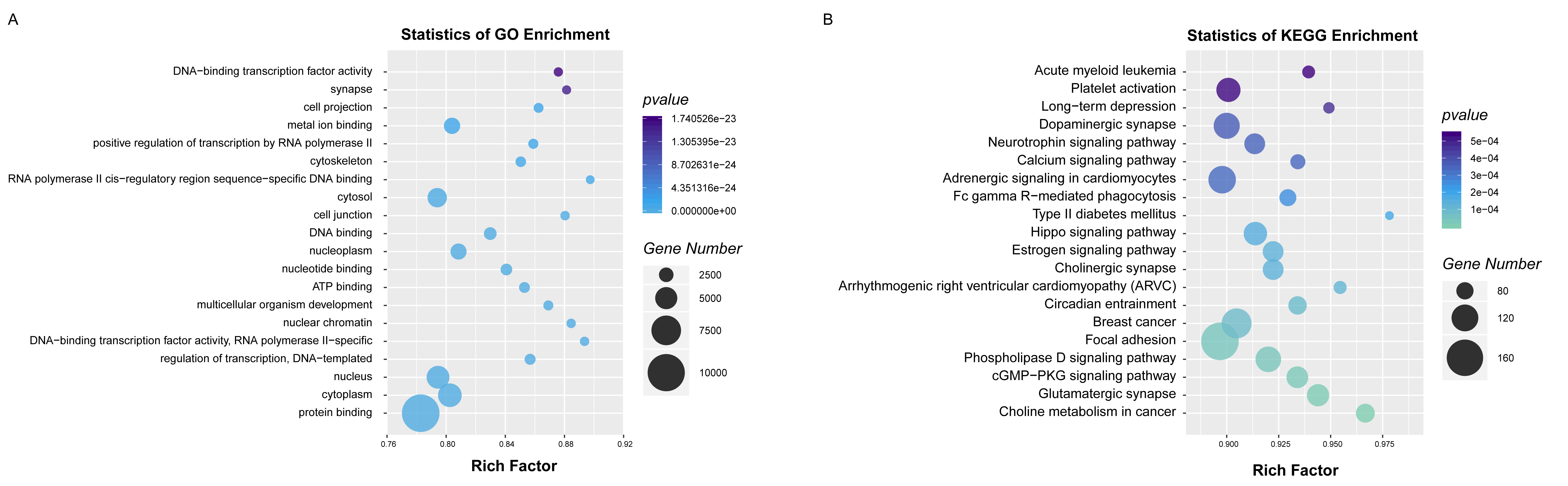

Supplement: Supplementary file 4 — Additional file 4: Fig. S4. GO and KEGG enrichment analysis of genes with DMRs. Dot plot showing biological process, molecular function, cellular component (A) and KEGG (B) with the significantly enriched terms. [file 13148_2023_1550_MOESM4_ESM.tif]

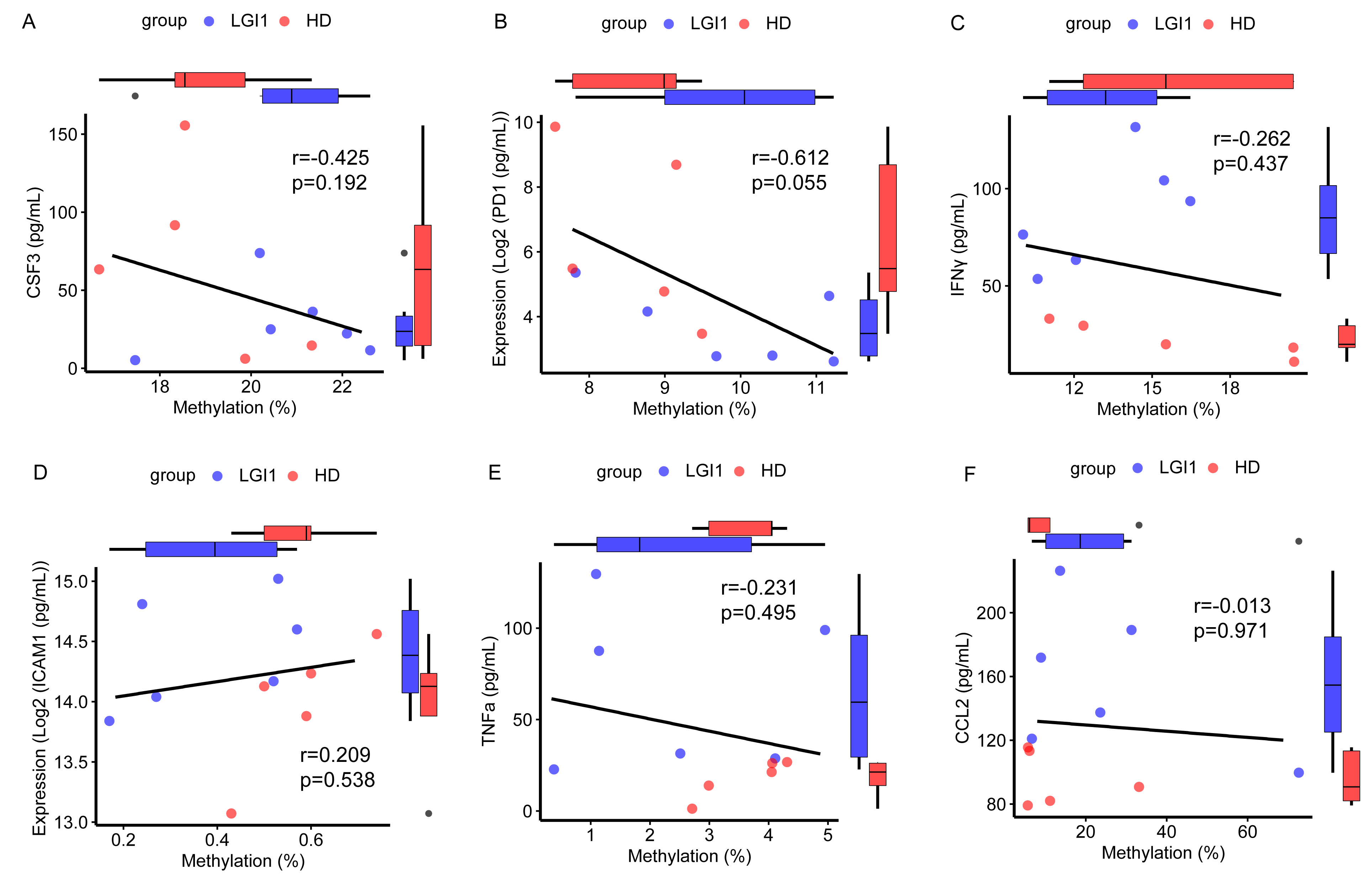

Supplement: Supplementary file 5 — Additional file 5: Fig. S5. The association of the methylation changes of DMRs within the promoter of 6 novel methylation genes with their concentration in serum. Scatterplots and box plots showing the correlation of methylation level of DMR within the promoter of CSF3 (A), PDCD1 (B), IFN-γ (C), ICAM1 (D), TNFɑ (E) and CCL2 (F) with their expression level in serum. [file 13148_2023_1550_MOESM5_ESM.tif]

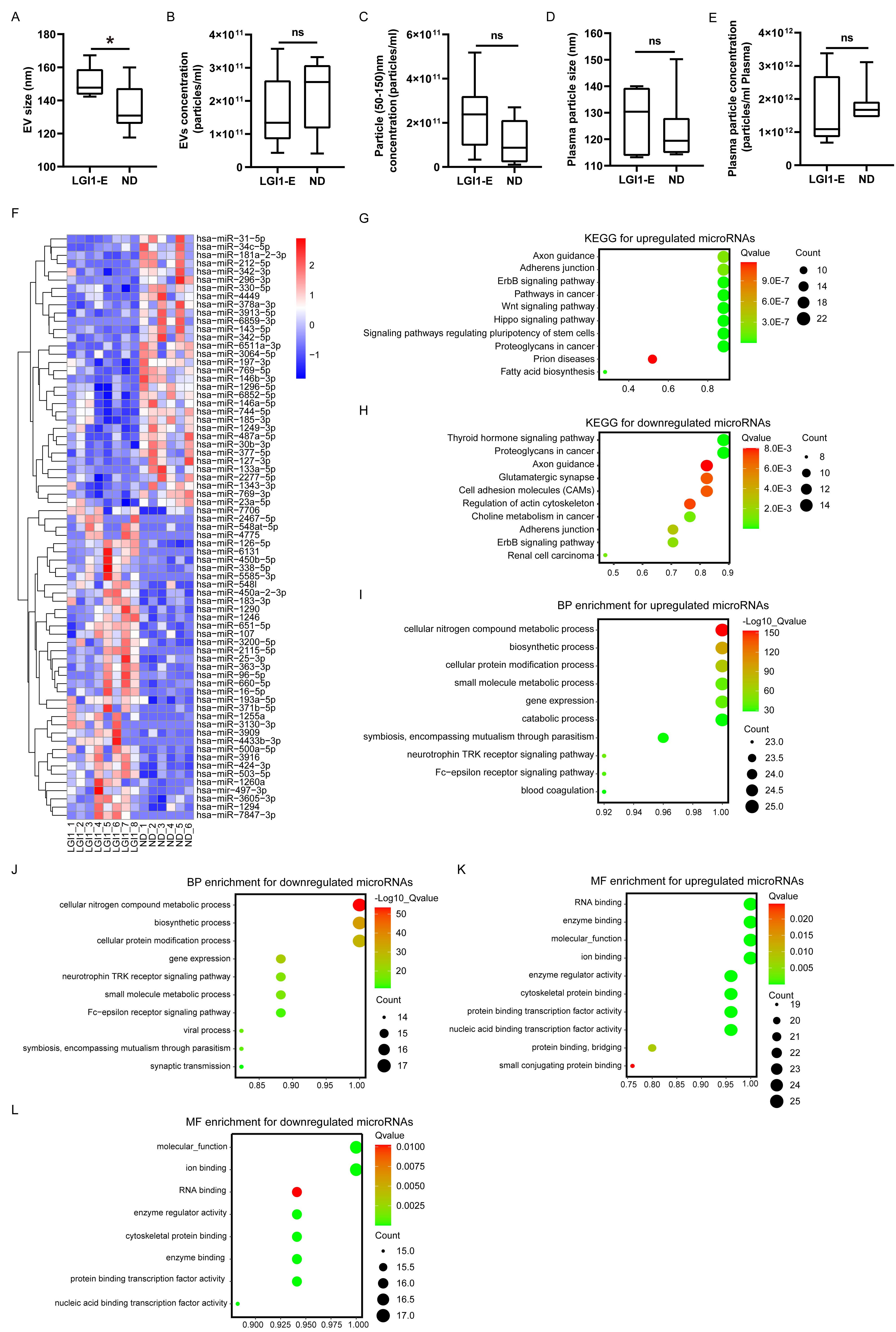

Supplement: Supplementary file 6 — Additional file 6: Fig. S6. The expression profiles of microRNAs in exosome and their potential functionality. NTA analysis the size of EVs (A), EV concentration (B), the concentration of particle within the diameter 50–150nm (C), particle size and concentration in plasma (D and E). (F) A heat map representing discrepant microRNA expression values in plasma exosome of LGI1 encephalitis patients compared with those of healthy control. (G) and (H) KEGG pathways for 38 significant upregulated exo-microRNAs and 33 downregulated exo-microRNAs. (I) and (J) BP enrichment for 38 significant upregulated exo-microRNAs and 33 downregulated exo-microRNAs. (K) and (L) MF enrichment for 38 significant upregulated exo-microRNAs and 33 downregulated exo-microRNAs. LGI1-E, LGI1 encephalitis; ND, normal donor. [file 13148_2023_1550_MOESM6_ESM.tif]

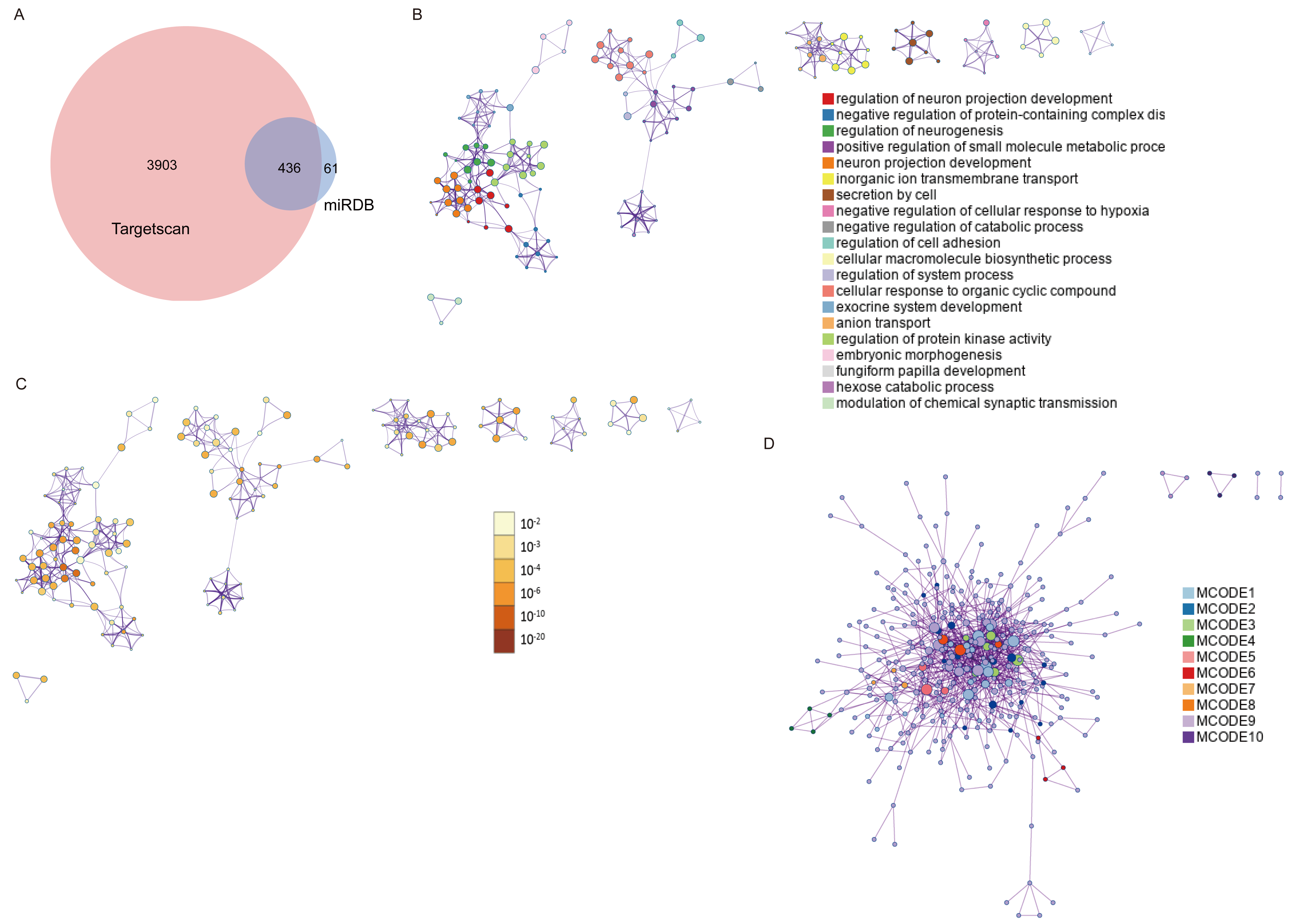

Supplement: Supplementary file 7 — Additional file 7: Fig. S7. The potential BP enrichment for exo-miR-2467-5p. (A) The 436 overlapped genes targeted by miR-2467-5p were acquired through bioinformatic tools. (B) The potential BP of 436 overlapped genes using Metascape. (C) The genes in Fig. S7B were colored by their match P value. (D) The enriched cluster showing the interaction of 10 modules in PPI network as analyzed by Metascape. [file 13148_2023_1550_MOESM7_ESM.tif]

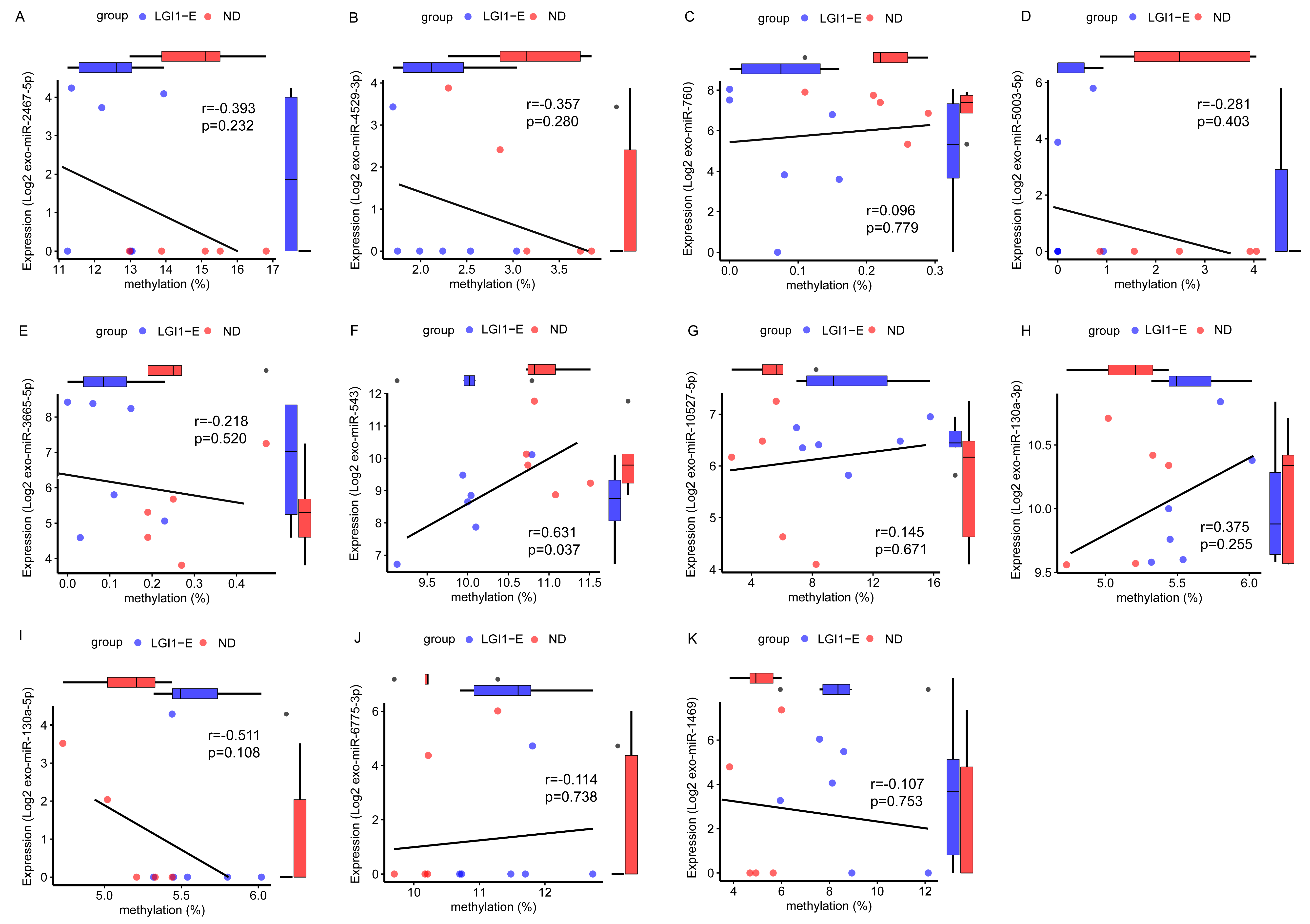

Supplement: Supplementary file 8 — Additional file 8: Fig. S8. The association of the methylation status of DMRs within the promoter of 13 differentially methylated microRNAs with their expression in plasma exosome. Scatterplots and box plots showing methylation level of DMR within the promoter of miR-2467-5p (A), miR-4529-3p (B), miR-760 (C), miR-5003-5p (D), miR-3665-5p (E), miR-543 (F), miR-10527-5p (G), miR-130a-3p (H), miR0130a-5p (I), miR-6775-3p (J) and miR-1469 (K) and their matched expression in exosome between LGI1-E patients and normal donors. LGI1-E LGI1 encephalitis; ND normal donor. [file 13148_2023_1550_MOESM8_ESM.tif]

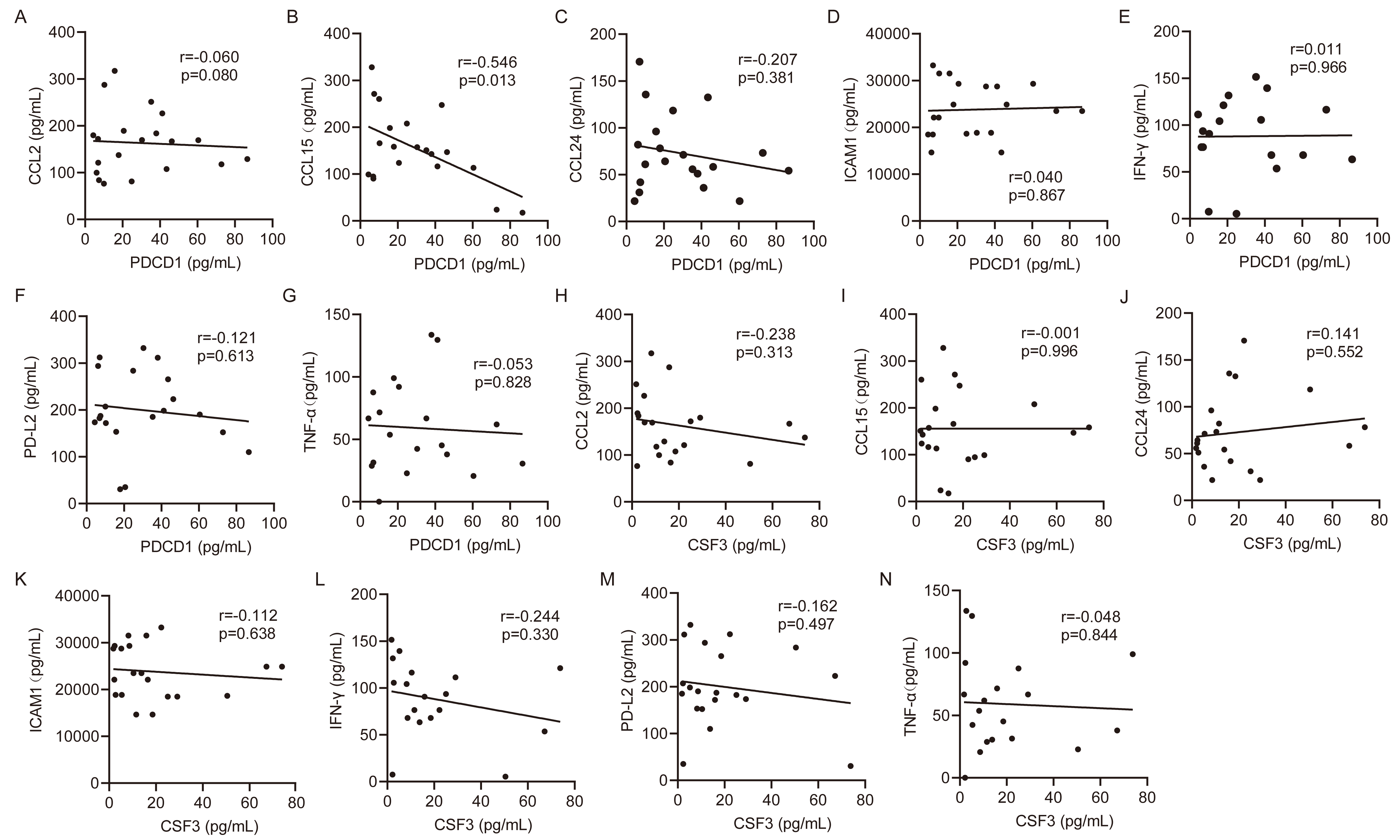

Supplement: Supplementary file 9 — Additional file 9: Fig. S9. The association of the level of plasma PDCD1 and CSF3 with multiple cytokines expression in plasma in LGI1 encephalitis patients. Scatterplots showing the association of PDCD1 level with the expression of CCL2 (A), CCL15 (B), CCL24 (C), ICAM1 (D), IFN-γ (E), PD-L2 (F) and TNF-ɑ (G) in LGI1 encephalitis. Scatterplots showing the association of CSF3 level in plasma with the expression of CCL2 (H), CCL15 (I), CCL24 (J), ICAM1 (K), IFN-γ (L), PD-L2 (M) and TNF-ɑ (N) in LGI1 encephalitis. [file 13148_2023_1550_MOESM9_ESM.tif]

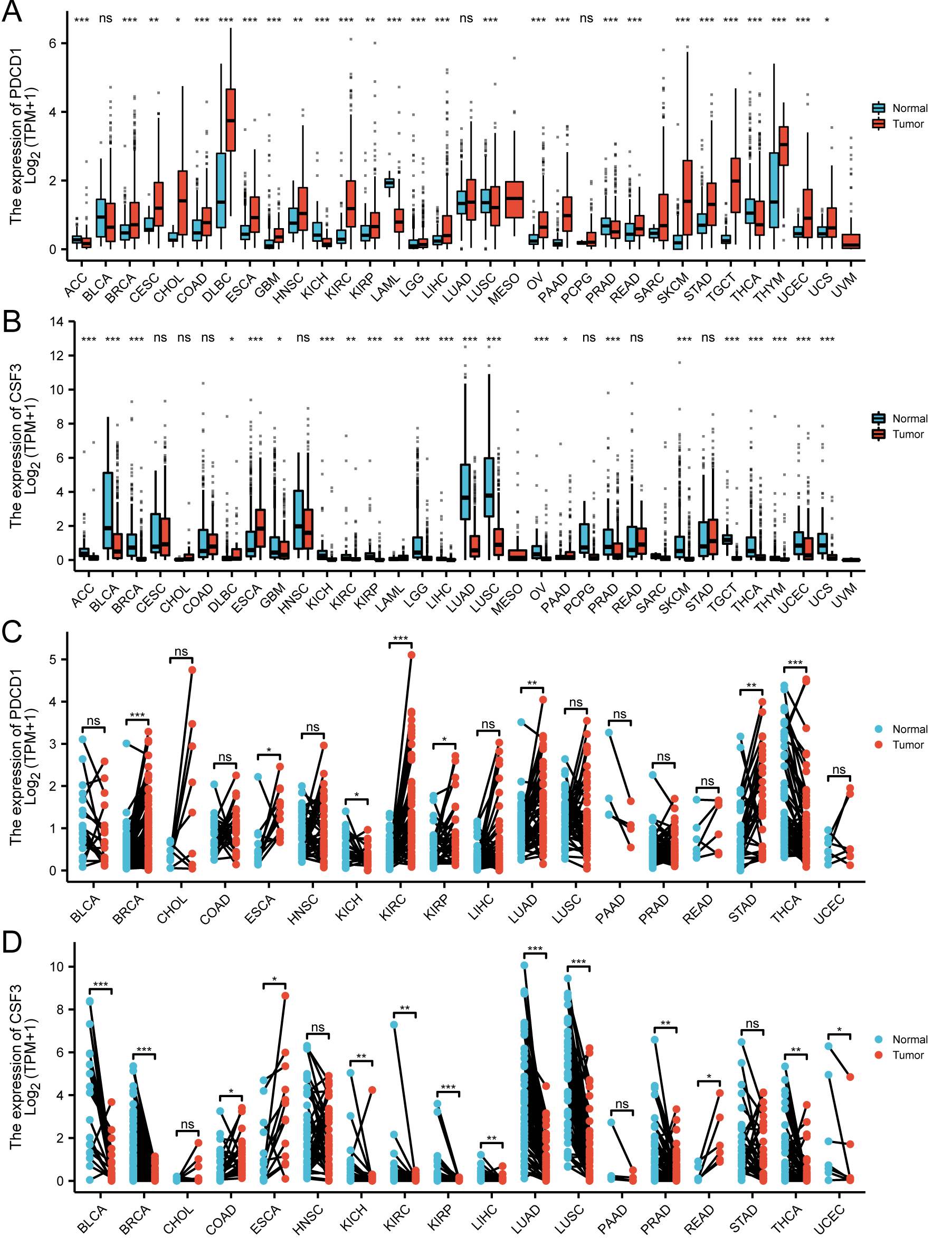

Supplement: Supplementary file 10 — Additional file 10: Fig. S10. PDCD1 and CSF3 expression in human cancers. A PDCD1 expression profiles in human cancers were compared with normal tissues using the TCGA dataset on the XIANTAO platform. B CSF3 expression profiles in human cancers were compared with normal tissues using the TCGA dataset. C PDCD1 expression profiles in human cancers were compared with the paired tissues using the TCGA dataset. D CSF3 expression profiles in human cancers were compared with the paired tissues using the TCGA dataset. *P < 0.05, **P < 0.01, ***P < 0.001. [file 13148_2023_1550_MOESM10_ESM.tif]

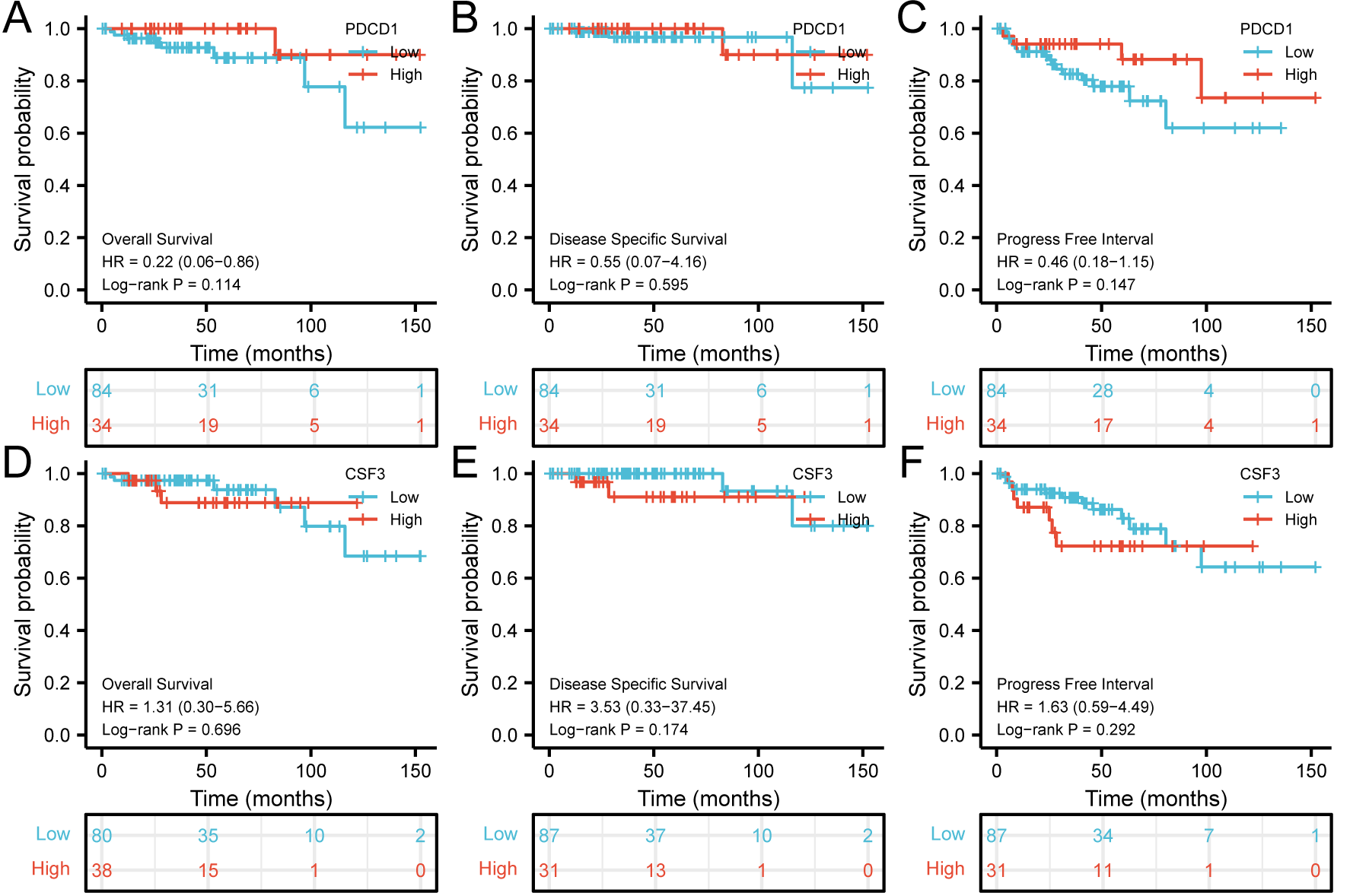

Supplement: Supplementary file 11 — Additional file 11: Fig. S11. The correlates of PDCD1 and CSF3 expression with survival outcome in thymoma patients. A–C Kaplan-Meier analysis of the correlation of PDCD1 expression with overall survival, disease-specific survival and progress free interval in thymoma patients. D–F Kaplan–Meier analysis of the correlation of CSF3 expression with overall survival, disease-specific survival and progress free interval in thymoma patients. [file 13148_2023_1550_MOESM11_ESM.tif]

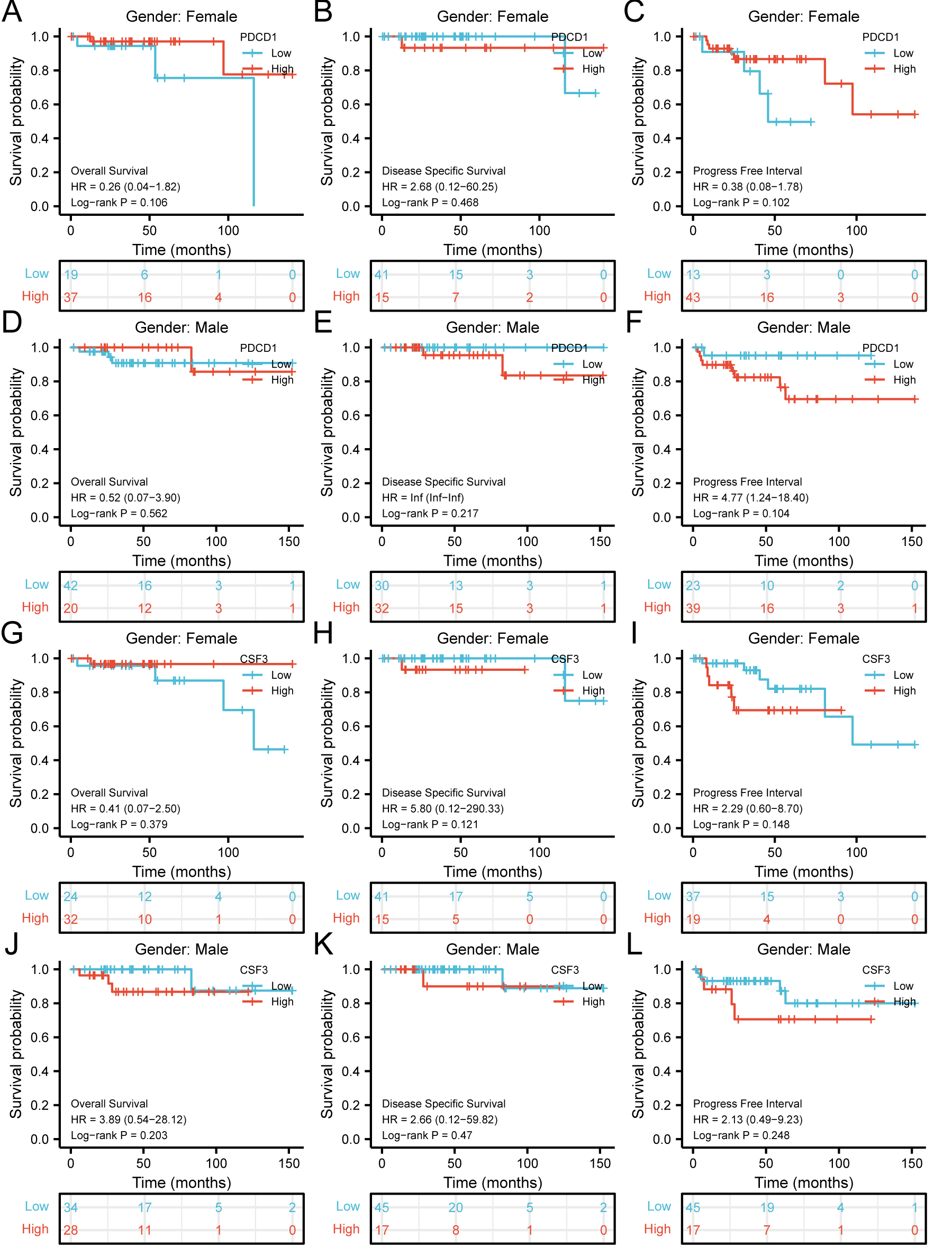

Supplement: Supplementary file 12 — Additional file 12: Fig. S12. The correlates of PDCD1 and CSF3 expression with survival outcome in female or male patients with thymoma. A–C Kaplan–Meier analysis of the correlation of PDCD1 expression with overall survival, disease-specific survival and progress free interval in female with thymoma. D–F The correlation of PDCD1 expression with overall survival, disease-specific survival and progress free interval in male with thymoma. G–I The correlation of CSF3 expression with overall survival, disease-specific survival and progress free interval in female with thymoma. J–L The correlation of PDCD1 expression with overall survival, disease-specific survival and progress free interval in male with thymoma. Case numbers in each group were listed at the bottom of the figure. [file 13148_2023_1550_MOESM12_ESM.tif]
